# Supplementary material for: A unifying framework for interpreting and predicting mutualistic systems
Source: Nat Commun. 2019 Jan 16;10:242. doi: 10.1038/s41467-018-08188-5 (PMC6335432; doi:10.1038/s41467-018-08188-5)
Supplement: Supplementary file 1 — Supplementary Information [file 41467_2018_8188_MOESM1_ESM.pdf]

# Supplementary Information for

## A unifying framework for interpreting and predicting mutualistic systems

Wu et al.

Supplementary Tables 1-2 (page 2-6)

Supplementary Figures 1-11 (page 7-19)

Supplementary Notes (page 20-43)

Supplementary References (page 44-45)

# Effect of cost (ε) is independent of partner density

| | | |
|---|---|---|
| $\boldsymbol{\frac{dX_1}{d\tau} = (1-\delta_1)X_1(1-X_1)}$ | | |
| 1. $\frac{dX_1}{d\tau} = (1-\delta\varepsilon\frac{1}{\beta X_2+1})X_1(1-X_1)$<br>$\frac{\beta X_0+1}{\varepsilon} > \delta$ | 2. $\frac{dX_1}{d\tau} = (1-\delta)\frac{1}{\varepsilon}X_1\left(1+\frac{\beta' X_2}{X_2+1/\beta}-X_1\right)$<br>Violates assumption d) and unbounded growth | 3. $\frac{dX_1}{d\tau} = (1-\delta\varepsilon)X_1(1-X_1)+\frac{\beta' X_2}{X_2+1/\beta}X_1$<br>Unbounded growth |
| 4. $\frac{dX_1}{d\tau} = (1-\delta\frac{1}{\beta X_2+1})X_1\left(\frac{1}{\varepsilon}-X_1\right)$<br>$\beta X_0+1 > \delta$ | 5. $\frac{dX_1}{d\tau} = (1-\delta)X_1\left(1+\frac{1}{\varepsilon}\frac{\beta' X_2}{X_2+1/\beta}-X_1\right)$<br>Violates assumption d) and unbounded growth | 6. $\frac{dX_1}{d\tau} = (1-\delta)X_1\left(\frac{1}{\varepsilon}-X_1\right)+\frac{\beta' X_2}{X_2+1/\beta}X_1$<br>Violates assumption d) and unbounded growth |
| 7. $\frac{dX_1}{d\tau} = (1-\delta\frac{1}{\beta X_2+1})X_1(1-X_1)-\varepsilon X_1$<br>$\left(\sqrt{\beta+1}-\sqrt{\beta\varepsilon}\right)^2 > \delta$ | 8. $\frac{dX_1}{d\tau} = (1-\delta)X_1\left(1+\frac{\beta' X_2}{X_2+1/\beta}-X_1\right)-\varepsilon X_1$<br>Violates assumption d) | 9. $\frac{dX_1}{d\tau} = (1-\delta)X_1(1-X_1)+\frac{1}{\varepsilon}\frac{\beta' X_2}{X_2+1/\beta}X_1$<br>Unbounded growth |
| $\boldsymbol{\frac{dX_1}{d\tau} = X_1((1-\delta_1)-X_1)}$ | | |
| 10. $\frac{dX_1}{d\tau} = \frac{1}{\varepsilon}\frac{\beta' X_2}{X_2+1/\beta}X_1((1-\delta)-X_1)$<br>Violates assumption d) | 11. $\frac{dX_1}{d\tau} = \frac{1}{\varepsilon}X_1\left((1-\delta\frac{1}{\beta X_2+1})-X_1\right)$<br>$\frac{(\beta+1)^2}{4\beta} > \delta$ | 12. $\frac{dX_1}{d\tau} = \frac{1}{\varepsilon}X_1(1-\delta-X_1)+\frac{\beta' X_2}{X_2+1/\beta}X_1$<br>Violates assumption d) |
| 13. $\frac{dX_1}{d\tau} = \frac{\beta' X_2}{X_2+1/\beta}X_1\left((1-\delta)\frac{1}{\varepsilon}-X_1\right)$<br>Violates assumption d) | 14. $\frac{dX_1}{d\tau} = X_1\left((1-\delta\frac{1}{\beta X_2+1})\frac{1}{\varepsilon}-X_1\right)$<br>Violates assumption d) | 15. $\frac{dX_1}{d\tau} = X_1\left(\frac{1}{\varepsilon}-\delta-X_1\right)+\frac{\beta' X_2}{X_2+1/\beta}X_1$<br>$\frac{\left(\sqrt{\beta\beta'\varepsilon}-\sqrt{\varepsilon}\right)^2+\beta}{\beta\varepsilon} > \delta$ |
| 16. $\frac{dX_1}{d\tau} = \frac{\beta' X_2}{X_2+1/\beta}X_1((1-\delta)-X_1)-\varepsilon X_1$<br>Violates assumption d) | 17. $\frac{dX_1}{d\tau} = X_1\left((1-\delta\frac{1}{\beta X_2+1})-X_1\right)-\varepsilon X_1$<br>$\frac{(\beta-\beta\varepsilon+1)^2}{4\beta} > \delta$ | 18. $\frac{dX_1}{d\tau} = X_1(1-\delta-X_1)+\frac{1}{\varepsilon}\frac{\beta' X_2}{X_2+1/\beta}X_1$<br>$\frac{\left(\sqrt{\beta'\beta}-\sqrt{\varepsilon}\right)^2+\beta\varepsilon}{\beta\varepsilon} > \delta$ |
| $\boldsymbol{\frac{dX_1}{d\tau} = X_1(1-X_1)-\delta_1 X_1}$ | | |
| 19. $\frac{dX_1}{d\tau} = \frac{1}{\varepsilon}\frac{\beta' X_2}{X_2+1/\beta}X_1(1-X_1)-\delta X_1$<br>$\frac{2\beta'-2\beta'\sqrt{\beta+1}+\beta\beta'}{\beta\varepsilon} > \delta$ | 20. $\frac{dX_1}{d\tau} = \frac{1}{\varepsilon}X_1\left(\frac{\beta' X_2}{X_2+1/\beta}-X_1\right)-\delta X_1$<br>$\frac{\left(\sqrt{\beta\beta'}-1\right)^2}{\beta\varepsilon} > \delta$ | 21. $\frac{dX_1}{d\tau} = \frac{1}{\varepsilon}X_1(1-X_1)-\delta\frac{1}{\beta X_2+1}X_1$<br>$\frac{(\beta+1)^2}{4\beta\varepsilon} > \delta$ |
| 22. $\frac{dX_1}{d\tau} = \frac{\beta' X_2}{X_2+1/\beta}X_1\left(\frac{1}{\varepsilon}-X_1\right)-\delta X_1$<br>$\frac{2\beta'\varepsilon-2\beta'\sqrt{\beta\varepsilon+\varepsilon^2}+\beta\beta'}{\beta\varepsilon} > \delta$ | 23. $\frac{dX_1}{d\tau} = X_1\left(\frac{1}{\varepsilon}\frac{\beta' X_2}{X_2+1/\beta}-X_1\right)-\delta X_1$<br>$\frac{\left(\sqrt{\beta\beta'}-\sqrt{\varepsilon}\right)^2+\beta\varepsilon}{\beta\varepsilon} > \delta$ | 24. $\frac{dX_1}{d\tau} = X_1\left(\frac{1}{\varepsilon}-X_1\right)-\delta\frac{1}{\beta X_2+1}X_1$<br>$\frac{(\beta+\varepsilon)^2}{4\beta\varepsilon^2} > \delta$ |
| 25. $\frac{dX_1}{d\tau} = \frac{\beta' X_2}{X_2+1/\beta}X_1(1-X_1)-\delta\varepsilon X_1$<br>$\frac{2\beta'-2\beta'\sqrt{\beta+1}+\beta\beta'}{\beta\varepsilon} > \delta$ | 26. $\frac{dX_1}{d\tau} = X_1\left(\frac{\beta' X_2}{X_2+1/\beta}-X_1\right)-\delta\varepsilon X_1$<br>$\frac{\left(\sqrt{\beta\beta'}-1\right)^2}{\beta\varepsilon} > \delta$ | 27. $\frac{dX_1}{d\tau} = X_1(1-X_1)-\delta\varepsilon\frac{1}{\beta X_2+1}X_1$<br>$\frac{(\beta+1)^2}{4\beta\varepsilon} > \delta$ |

# Sharing carrying capacity

| | | |
|---|---|---|
| $\boldsymbol{\frac{dX_1}{d\tau} = (1-\delta_1)X_1(1-X_1-X_2)}$ | | |
| 28. $\frac{dX_1}{d\tau} = (1-\delta\varepsilon\frac{1}{\beta X_2+1})X_1(1-X_1-X_2)$<br>$\frac{\beta X_0+1}{\varepsilon} > \delta$ | 29. $\frac{dX_1}{d\tau} = (1-\delta)\frac{1}{\varepsilon}X_1\left(\frac{\beta' X_2}{X_2+1/\beta} - X_1 - X_2\right)$<br>Violates assumption d) and unbounded growth | 30. $\frac{dX_1}{d\tau} = (1-\delta\varepsilon)X_1(1-X_1-X_2) + \frac{\beta' X_2}{X_2+1/\beta}X_1$<br>Unbounded growth |
| 31. $\frac{dX_1}{d\tau} = (1-\delta\frac{1}{\beta X_2+1})X_1\left(\frac{1}{\varepsilon} - X_1 - X_2\right)$<br>$\beta X_0 + 1 > \delta$ | 32. $\frac{dX_1}{d\tau} = (1-\delta)X_1\left(\frac{1}{\varepsilon}\frac{\beta' X_2}{X_2+1/\beta} - X_1 - X_2\right)$<br>Violates assumption d) and unbounded growth | 33. $\frac{dX_1}{d\tau} = (1-\delta)X_1\left(\frac{1}{\varepsilon} - X_1 - X_2\right) + \frac{\beta' X_2}{X_2+1/\beta}X_1$<br>Violates assumption d) and unbounded growth |
| 34. $\frac{dX_1}{d\tau} = (1-\delta\frac{1}{\beta X_2+1})X_1(1-X_1-X_2) - \varepsilon X_1$<br>$\frac{1}{2}\left(\sqrt{\beta+2} - \sqrt{\beta\varepsilon}\right)^2 > \delta$ | 35. $\frac{dX_1}{d\tau} = (1-\delta)X_1\left(\frac{\beta' X_2}{X_2+1/\beta} - X_1 - X_2\right) - \varepsilon X_1$<br>Violates assumption d) | 36. $\frac{dX_1}{d\tau} = (1-\delta)X_1(1-X_1-X_2) + \frac{1}{\varepsilon}\frac{\beta' X_2}{X_2+1/\beta}X_1$<br>Unbounded growth |
| $\boldsymbol{\frac{dX_1}{d\tau} = X_1((1-\delta_1) - X_1 - X_2)}$ | | |
| 37. $\frac{dX_1}{d\tau} = \frac{1}{\varepsilon}\frac{\beta' X_2}{X_2+1/\beta}X_1((1-\delta) - X_1 - X_2)$<br>Violates assumption d) | 38. $\frac{dX_1}{d\tau} = \frac{1}{\varepsilon}X_1\left((1-\delta\frac{1}{\beta X_2+1}) - X_1 - X_2\right)$<br>$\frac{(\beta+2)^2}{8\beta} > \delta$ | 39. $\frac{dX_1}{d\tau} = \frac{1}{\varepsilon}X_1(1-\delta-X_1-X_2) + \frac{\beta' X_2}{X_2+1/\beta}X_1$<br>Violates assumption d) |
| 40. $\frac{dX_1}{d\tau} = \frac{\beta' X_2}{X_2+1/\beta}X_1\left((1-\delta)\frac{1}{\varepsilon} - X_1 - X_2\right)$<br>Violates assumption d) | 41. $\frac{dX_1}{d\tau} = X_1\left((1-\delta\frac{1}{\beta X_2+1})\frac{1}{\varepsilon} - X_1 - X_2\right)$<br>Violates assumption d) | 42. $\frac{dX_1}{d\tau} = X_1\left(\frac{1}{\varepsilon} - \delta - X_1 - X_2\right) + \frac{\beta' X_2}{X_2+1/\beta}X_1$<br>$\frac{\varepsilon\left(\sqrt{\beta\beta'} - \sqrt{2}\right)^2 + \beta}{\beta\varepsilon} > \delta$ |
| 43. $\frac{dX_1}{d\tau} = \frac{\beta' X_2}{X_2+1/\beta}X_1((1-\delta) - X_1 - X_2) - \varepsilon X_1$<br>Violates assumption d) | 44. $\frac{dX_1}{d\tau} = X_1\left((1-\delta\frac{1}{\beta X_2+1}) - X_1 - X_2\right) - \varepsilon X_1$<br>$\frac{(\beta+2-\beta\varepsilon)^2}{8\beta} > \delta$ | 45. $\frac{dX_1}{d\tau} = X_1(1-\delta-X_1-X_2) + \frac{1}{\varepsilon}\frac{\beta' X_2}{X_2+1/\beta}X_1$<br>$\frac{\left(\sqrt{\beta'\beta} - \sqrt{2\varepsilon}\right)^2 + \beta\varepsilon}{\beta\varepsilon} > \delta$ |
| $\boldsymbol{\frac{dX_1}{d\tau} = X_1(1-X_1-X_2) - \delta_1 X_1}$ | | |
| 46. $\frac{dX_1}{d\tau} = \frac{1}{\varepsilon}\frac{\beta' X_2}{X_2+1/\beta}X_1(1-X_1-X_2) - \delta X_1$<br>$\frac{4\beta' - 2\beta'\sqrt{2\beta+4} + \beta\beta'}{\beta\varepsilon} > \delta$ | 47. $\frac{dX_1}{d\tau} = \frac{1}{\varepsilon}X_1\left(\frac{\beta' X_2}{X_2+1/\beta} - X_1 - X_2\right) - \delta X_1$<br>$\frac{\left(\sqrt{\beta\beta'} - \sqrt{2}\right)^2}{\beta\varepsilon} > \delta$ | 48. $\frac{dX_1}{d\tau} = \frac{1}{\varepsilon}X_1(1-X_1-X_2) - \delta\frac{1}{\beta X_2+1}X_1$<br>$\frac{(\beta+2)^2}{8\beta\varepsilon} > \delta$ |
| 49. $\frac{dX_1}{d\tau} = \frac{\beta' X_2}{X_2+1/\beta}X_1\left(\frac{1}{\varepsilon} - X_1 - X_2\right) - \delta X_1$<br>$\frac{4\beta'\varepsilon - 2\beta'\sqrt{2\beta\varepsilon+4\varepsilon^2} + \beta\beta'}{\beta\varepsilon} > \delta$ | 50. $\frac{dX_1}{d\tau} = X_1\left(\frac{1}{\varepsilon}\frac{\beta' X_2}{X_2+1/\beta} - X_1 - X_2\right) - \delta X_1$<br>$\frac{\left(\sqrt{\beta\beta'} - \sqrt{2\varepsilon}\right)^2}{\beta\varepsilon} > \delta$ | 51. $\frac{dX_1}{d\tau} = X_1\left(\frac{1}{\varepsilon} - X_1 - X_2\right) - \delta\frac{1}{\beta X_2+1}X_1$<br>$\frac{(\beta+2\varepsilon)^2}{8\beta\varepsilon^2} > \delta$ |
| 52. $\frac{dX_1}{d\tau} = \frac{\beta' X_2}{X_2+1/\beta}X_1(1-X_1-X_2) - \delta\varepsilon X_1$<br>$\frac{4\beta' - 2\beta'\sqrt{2\beta+4} + \beta\beta'}{\beta\varepsilon} > \delta$ | 53. $\frac{dX_1}{d\tau} = X_1\left(\frac{\beta' X_2}{X_2+1/\beta} - X_1 - X_2\right) - \delta\varepsilon X_1$<br>$\frac{\left(\sqrt{\beta\beta'} - \sqrt{2}\right)^2}{\beta\varepsilon} > \delta$ | 54. $\frac{dX_1}{d\tau} = X_1(1-X_1-X_2) - \delta\varepsilon\frac{1}{\beta X_2+1}X_1$<br>$\frac{(\beta+2)^2}{8\beta\varepsilon} > \delta$ |

| | | |
|---|---|---|
| **Effect of cost (ε) increases with partner density** | | |
| $\boldsymbol{\frac{dX_1}{d\tau} = (1-\delta_1)X_1(1-X_1)}$ | | |
| 55. $\frac{dX_1}{d\tau} = (1-\delta(1+\varepsilon X_2)\frac{1}{\beta X_2+1})X_1(1-X_1)$<br>$\frac{\beta X_0+1}{\varepsilon X_0+1} > \delta$ | 56. $\frac{dX_1}{d\tau} = (1-\delta)\frac{1}{(1+\varepsilon X_2)}X_1\left(\frac{\beta' X_2}{X_2+1/\beta} - X_1\right)$<br>Violates assumption d) and unbounded growth | 57. $\frac{dX_1}{d\tau} = (1-\delta(1+\varepsilon X_2))X_1(1-X_1) + \frac{\beta' X_2}{X_2+1/\beta}X_1$<br>Unbounded growth |
| 58. $\frac{dX_1}{d\tau} = (1-\delta\frac{1}{\beta X_2+1})X_1\left(\frac{1}{(1+\varepsilon X_2)} - X_1\right)$<br>$\beta X_0+1 > \delta$ | 59. $\frac{dX_1}{d\tau} = (1-\delta)X_1\left(\frac{1}{(1+\varepsilon X_2)}\frac{\beta' X_2}{X_2+1/\beta} - X_1\right)$<br>Violates assumption d) and unbounded growth | 60. $\frac{dX_1}{d\tau} = (1-\delta)X_1\left(\frac{1}{(1+\varepsilon X_2)} - X_1\right) + \frac{\beta' X_2}{X_2+1/\beta}X_1$<br>Violates assumption d) and unbounded growth |
| 61. $\frac{dX_1}{d\tau} = (1-\delta\frac{1}{\beta X_2+1})X_1(1-X_1) - (\varepsilon X_2)X_1$<br>$\left(\sqrt{\beta\varepsilon} - \sqrt{(\varepsilon+1)(\beta+1)}\right)^2 > \delta$ | 62. $\frac{dX_1}{d\tau} = (1-\delta)X_1\left(\frac{\beta' X_2}{X_2+1/\beta} - X_1\right) - (\varepsilon X_2)X_1$<br>Violates assumption d) | 63. $\frac{dX_1}{d\tau} = (1-\delta)X_1(1-X_1) + \frac{1}{(1+\varepsilon X_2)}\frac{\beta' X_2}{X_2+1/\beta}X_1$<br>Unbounded growth |
| $\boldsymbol{\frac{dX_1}{d\tau} = X_1((1-\delta_1)-X_1)}$ | | |
| 64. $\frac{dX_1}{d\tau} = \frac{1}{(1+\varepsilon X_2)}\frac{\beta' X_2}{X_2+1/\beta}X_1((1-\delta)-X_1)$<br>Violates assumption d) | 65. $\frac{dX_1}{d\tau} = \frac{1}{(1+\varepsilon X_2)}X_1\left((1-\delta\frac{1}{\beta X_2+1}) - X_1\right)$<br>$\frac{(\beta+1)^2}{4\beta} > \delta$ | 66. $\frac{dX_1}{d\tau} = \frac{1}{(1+\varepsilon X_2)}X_1(1-\delta-X_1) + \frac{\beta' X_2}{X_2+1/\beta}X_1$<br>Violates assumption d) |
| 67. $\frac{dX_1}{d\tau} = \frac{\beta' X_2}{X_2+1/\beta}X_1\left((1-\delta)\frac{1}{(1+\varepsilon X_2)} - X_1\right)$<br>Violates assumption d) | 68. $\frac{dX_1}{d\tau} = X_1\left((1-\delta\frac{1}{\beta X_2+1})\frac{1}{(1+\varepsilon X_2)} - X_1\right)$<br>Violates assumption d) | 69. $\frac{dX_1}{d\tau} = X_1\left(\frac{1}{1+\varepsilon X_2} - \delta - X_1\right) + \frac{\beta' X_2}{X_2+1/\beta}X_1$<br>$f(\beta,\beta',\varepsilon) > \delta$ |
| 70. $\frac{dX_1}{d\tau} = \frac{\beta' X_2}{X_2+1/\beta}X_1((1-\delta)-X_1) - (\varepsilon X_2)X_1$<br>Violates assumption d) | 71. $\frac{dX_1}{d\tau} = X_1\left((1-\delta\frac{1}{\beta X_2+1}) - X_1\right) - (\varepsilon X_2)X_1$<br>$\frac{(\beta+\varepsilon+1)^2}{4\beta(\varepsilon+1)} > \delta$ | 72. $\frac{dX_1}{d\tau} = X_1(1-\delta-X_1) + \frac{1}{(1+\varepsilon X_2)}\frac{\beta' X_2}{X_2+1/\beta}X_1$<br>$f(\beta,\beta',\varepsilon) > \delta$ |
| $\boldsymbol{\frac{dX_1}{d\tau} = X_1(1-X_1) - \delta_1 X_1}$ | | |
| 73. $\frac{dX_1}{d\tau} = \frac{1}{(1+\varepsilon X_2)}\frac{\beta' X_2}{X_2+1/\beta}X_1(1-X_1) - \delta X_1$<br>$\frac{\beta\beta'\left(\sqrt{(\beta+1)} - \sqrt{(\varepsilon+1)}\right)^2}{(\beta-\varepsilon)^2} > \delta$ | 74. $\frac{dX_1}{d\tau} = \frac{1}{(1+\varepsilon X_2)}X_1\left(\frac{\beta' X_2}{X_2+1/\beta} - X_1\right) - \delta X_1$<br>$\frac{\left(\sqrt{\beta^2\beta'} - \sqrt{\beta-\varepsilon+\beta\beta'\varepsilon}\right)^2}{(\beta-\varepsilon)^2} > \delta$ | 75. $\frac{dX_1}{d\tau} = \frac{1}{(1+\varepsilon X_2)}X_1(1-X_1) - \delta\frac{1}{\beta X_2+1}X_1$<br>$\frac{\left(\sqrt{\beta} - \sqrt{(1+\varepsilon)(\beta-\varepsilon)}\right)^2}{\varepsilon^2} + 1 > \delta$ |
| 76. $\frac{dX_1}{d\tau} = \frac{\beta' X_2}{X_2+1/\beta}X_1\left(\frac{1}{(1+\varepsilon X_2)} - X_1\right) - \delta X_1$<br>$f(\beta,\beta',\varepsilon) > \delta$ | 77. $\frac{dX_1}{d\tau} = X_1\left(\frac{1}{(1+\varepsilon X_2)}\frac{\beta' X_2}{X_2+1/\beta} - X_1\right) - \delta X_1$<br>$f(\beta,\beta',\varepsilon) > \delta$ | 78. $\frac{dX_1}{d\tau} = X_1\left(\frac{1}{(1+\varepsilon X_2)} - X_1\right) - \delta\frac{1}{\beta X_2+1}X_1$<br>$f(\beta,\beta',\varepsilon) > \delta$ |
| 79. $\frac{dX_1}{d\tau} = \frac{\beta' X_2}{X_2+1/\beta}X_1(1-X_1) - \delta(1+\varepsilon X_2)X_1$<br>$\frac{\beta\beta'\left(\sqrt{(\beta+1)} - \sqrt{(\varepsilon+1)}\right)^2}{(\beta-\varepsilon)^2} > \delta$ | 80. $\frac{dX_1}{d\tau} = X_1\left(\frac{\beta' X_2}{X_2+1/\beta} - X_1\right) - \delta(1+\varepsilon X_2)X_1$<br>$\frac{\left(\sqrt{\beta^2\beta'} - \sqrt{\beta-\varepsilon+\beta\beta'\varepsilon}\right)^2}{(\beta-\varepsilon)^2} > \delta$ | 81. $\frac{dX_1}{d\tau} = X_1(1-X_1) - \delta(1+\varepsilon X_2)\frac{1}{\beta X_2+1}X_1$<br>$\frac{\left(\sqrt{\beta} - \sqrt{(1+\varepsilon)(\beta-\varepsilon)}\right)^2}{\varepsilon^2} + 1 > \delta$ |

**Supplementary Table 1**: Diverse coexistence criteria derived from a set of 81 mutualism models. The formulations are based on the locations of benefit ($\beta, \beta'$), cost ($\varepsilon$), and stress ($\delta$) in logistic growth equations (see Supplementary Note 2 for detailed rationale and method). Each $3 \times 3$ block represents different locations of the $\delta$ term. Each column and row represent a location of the benefit and cost term, respectively. Because we assume symmetry between the two populations, only the equation representing $\mathrm{X}_1$ is shown for simplicity of presentation. Models in the first table assume cost is independent of partner density. In natural mutualism systems, mutualists can also compete for resources, so the third table contains models that describe the two partners sharing the same carrying capacity. In addition, cost can also scale with partner densities, so models in the second table add density-dependent cost as linear dependencies. Models highlighted in grey either violate model assumption or generate unbounded growth, or both. The rest of the 48 models satisfy the 4 model assumptions and have bounded growth. For criteria that have a long left-hand side are written as $f(\beta, \beta', \varepsilon) > \delta$. The specific forms of $f(\beta, \beta', \varepsilon) > \delta$ can be found in the Supplementary Software. The Supplementary Software also includes all 81 models in this table and the process of testing assumptions, calculating and verifying the above criteria.

| Adding complexities | Inequality | Parameters |
|---|---|---|
| **Base model criterion** | 21. $\frac{(\beta+1)^2}{4\beta\varepsilon} > \delta$ | $\beta, \varepsilon$ |
| **Include competition** | 82. $\frac{(\beta+a+1)^2}{4\beta\varepsilon(a+1)} > \delta$ | $\beta, \varepsilon, a$ |
| **Include initial density** | 83. $\frac{1}{\varepsilon}(1-x_0)x_0\beta + \frac{1}{\varepsilon}(1-x_0) > \delta$ | $\beta, \varepsilon, x_0$ |
| **Include asymmetry** | 84. $\frac{\rho(\beta_1+1/\theta_1+1)^2}{4\beta_1\varepsilon_1(\rho/\theta_1+\theta_2)} > \delta_1$ <br> $\theta_1 = \frac{\beta_2}{\beta_1}, \theta_2 = \frac{\varepsilon_2\delta_2}{\varepsilon_1\delta_1}$ | $\beta, \varepsilon, \rho, \theta_1, \theta_2$ |
| **Include turnover rate** | 85. $\frac{(\beta(1-\varepsilon\delta_0)+2)^2}{4\beta\varepsilon}\left(\frac{\rho}{\rho+1}\right) > \delta$ | $\beta, \varepsilon, \rho, \delta_0$ |
| **N-mutualist system** | 86. $\frac{(\beta+\frac{n}{n-1})^2}{4\beta\varepsilon\frac{n}{n-1}} > \delta$ | $\beta, \varepsilon, n$ |

**Supplementary Table 2**: Increasing model complexity increases criterion complexity. The base model is identical to model 21 in Supplementary Table 1. Number 82-86 and their corresponding criteria are obtained by relaxing assumptions in model 21. Including the 48 models in Supplementary Table 1, we in total obtained coexistence criteria for 52 models. Note that number 21 and number 83 used the same model structure. The detailed models and criterion derivations are shown in Supplementary Note 3. Also see Supplementary Software for the above models and the process of calculating and verifying the corresponding criteria.

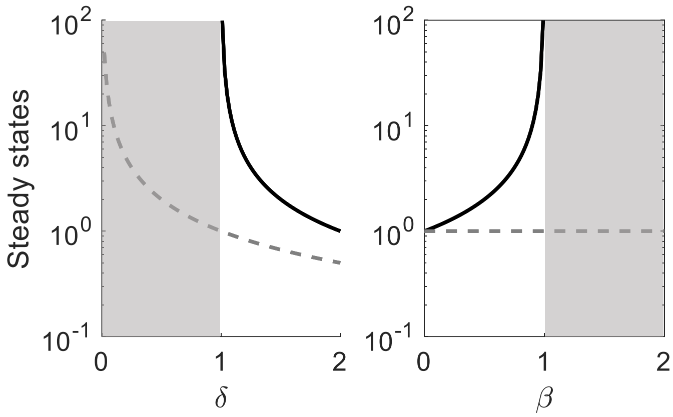


**Supplementary Figure 1**: **Typical bifurcation diagrams of models that create unbounded growth.** Previously-developed general coexistence criteria describe the boundary between stable coexistence (white regions) and unbounded growth (grey regions). Solid lines represent stable steady state for coexistence. Grey dashed lines represent steady states of a mutualist with the absence of its partner. This diagram is generated using model:

$$\frac{dX_1}{d\tau} = X_1(1 - \delta X_1) + \beta X_2 X_1$$

$$\frac{dX_2}{d\tau} = X_2(1 - \delta X_2) + \beta X_1 X_2$$

The left panel is generated with $\beta = 1$ and the right panel is generated with $\delta = 1$.

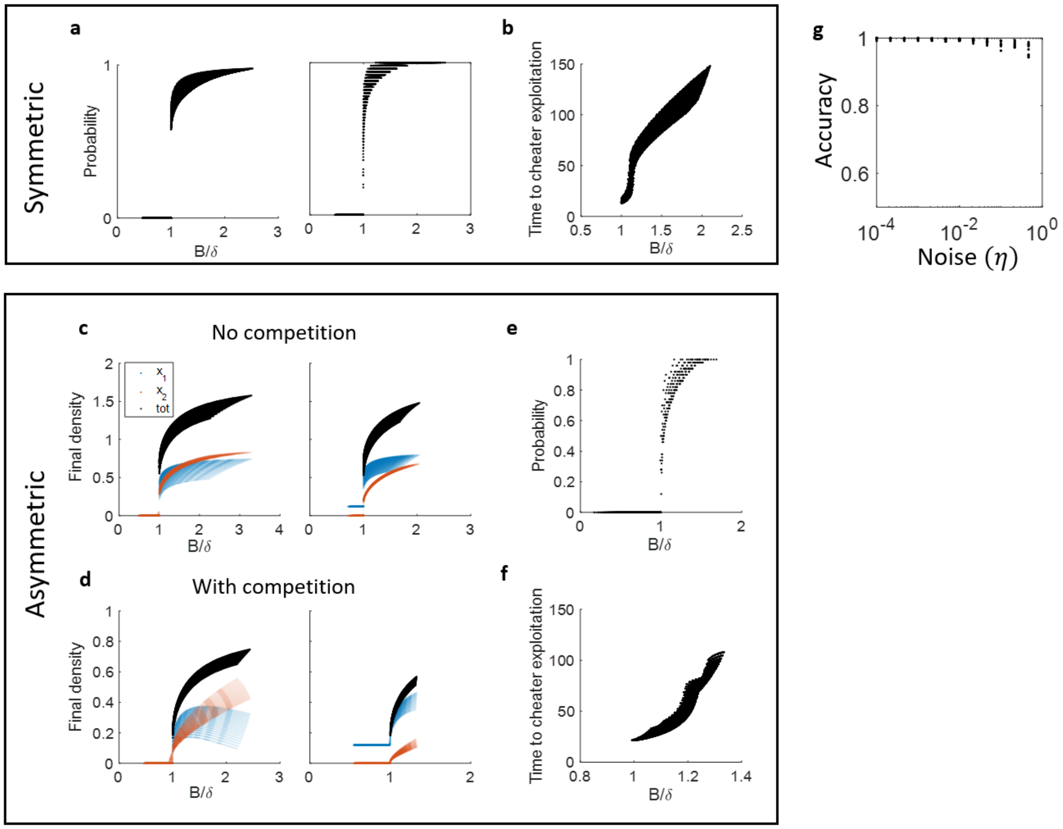


**Supplementary Figure 2**: **The predictor $B/\delta$ is a general metric for quantitative predictions**. All the traces are comprised of individual dots. Each dot represents a result from one set of model parameter. See Supplementary Note 4.1 for models and parameter values used in generating this panel a-f.

**a.** Mutualistic systems have initial-density-dependent coexistence. In the left panel, we assume the same initial density of both populations that is a uniform random variable. In the right panel, we varied the ratio of the two initial densities and kept the total initial density constant. In both cases, $B/\delta$ is predictive of coexistence probability.

**b.** When cheaters can arise in a system, $B/\delta$ is predictive of the time duration the system can persist before cheater exploitation.

**c.** An asymmetric mutualistic system can be either obligatory (left panel), where both populations extinct when $B/\delta < 1$ or facultative (right panel), where one population can persist even when $B/\delta < 1$. The black dots represent total density and the two colors represent the total density of the two partners. The same representations are used in panel d.

**d.** The predictive power of $B/\delta$ also holds for asymmetric systems that share carrying capacity.

**e.** $B/\delta$ is predictive of probability of coexistence for asymmetric systems.

**f.** The predictive power of $B/\delta$ for resistance to cheater exploitation also holds for asymmetric systems. The accuracy of criterion is robustly maintained after addition of extrinsic Gaussian noise. The x axis indicates the standard deviation of the Gaussian noise. Note that the noise is significant considering the maximum total density of the system is 1. Please see Supplementary Note 4.2 for the model and simulation details.

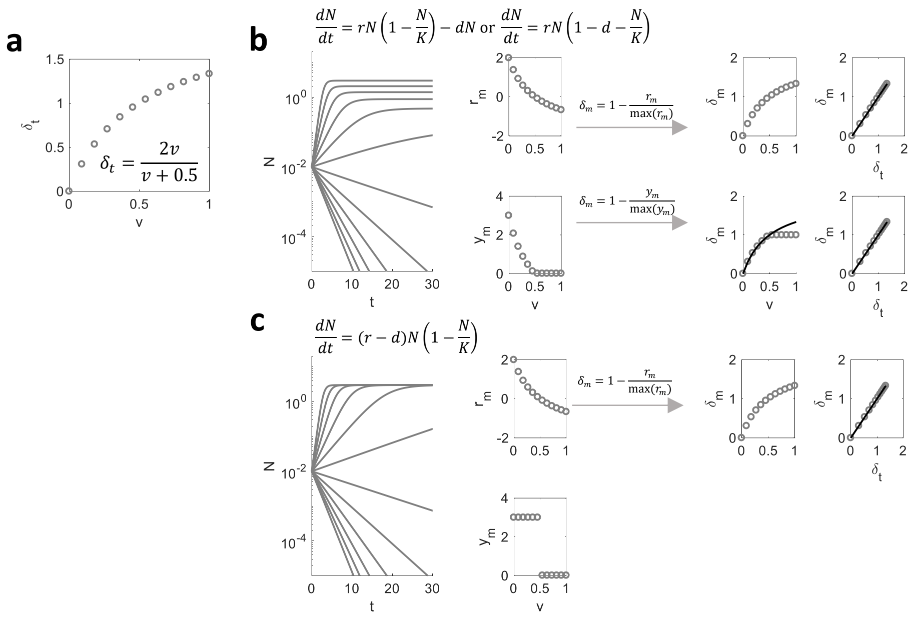


**Supplementary Figure 3: General methods for quantifying $\delta$.** $\delta$ can be measured using either growth rate or final population size with the absence of partner.

- **a.** We simulated the growth of a population that is modulated by a death term $d$. Stress $\delta$ is simply $d$ normalized by growth rate $r$. In the simulations, the true $\delta$ ($\delta_t$) depends on an independent variable $v$.
- **b.** One type of model structure has $\delta$ modulating both growth rate ($r$) and yield ($y$, final density). $N$ is the population size and $K$ is the carrying capacity. Based on the growth curve or final population size, measurements of growth rate ($r_m$) and yield ($y_m$) can be obtained by varying $v$. The $\delta$ measurement ($\delta_m$) can be calculated by $1 - \frac{r_m}{\max(r_m)}$ or $1 - \frac{y_m}{\max(y_m)}$. Note that since $y_m$ is always non-negative, $1 - \frac{y_m}{\max(y_m)}$ is always $\leq 1$, but $\delta$ can be greater than 1 when the population experiences negative growth. To address this discrepancy, extrapolation of $\delta_m$ is needed to capture death rate (the black trace in the $\delta_m$ versus $v$ plot).
- **c.** In another type of model formulation, $\delta$ only modulates growth rate. Simulation shows that $y_m$ either equals to carrying capacity or 0. Thus, in this case, only $r_m$ can be used to calculate $\delta$. The right-most panels show the comparison between the measured $\delta$ ($\delta_m$) and true $\delta$ ($\delta_t$). In all cases, $\delta_m$ is a good approximation of $\delta_t$ (the solid black line represents $\delta_m = \delta_t$). To summarize, $\delta$ can be obtained using either growth rate or yield of a mutualistic partner with the absence of its partner.

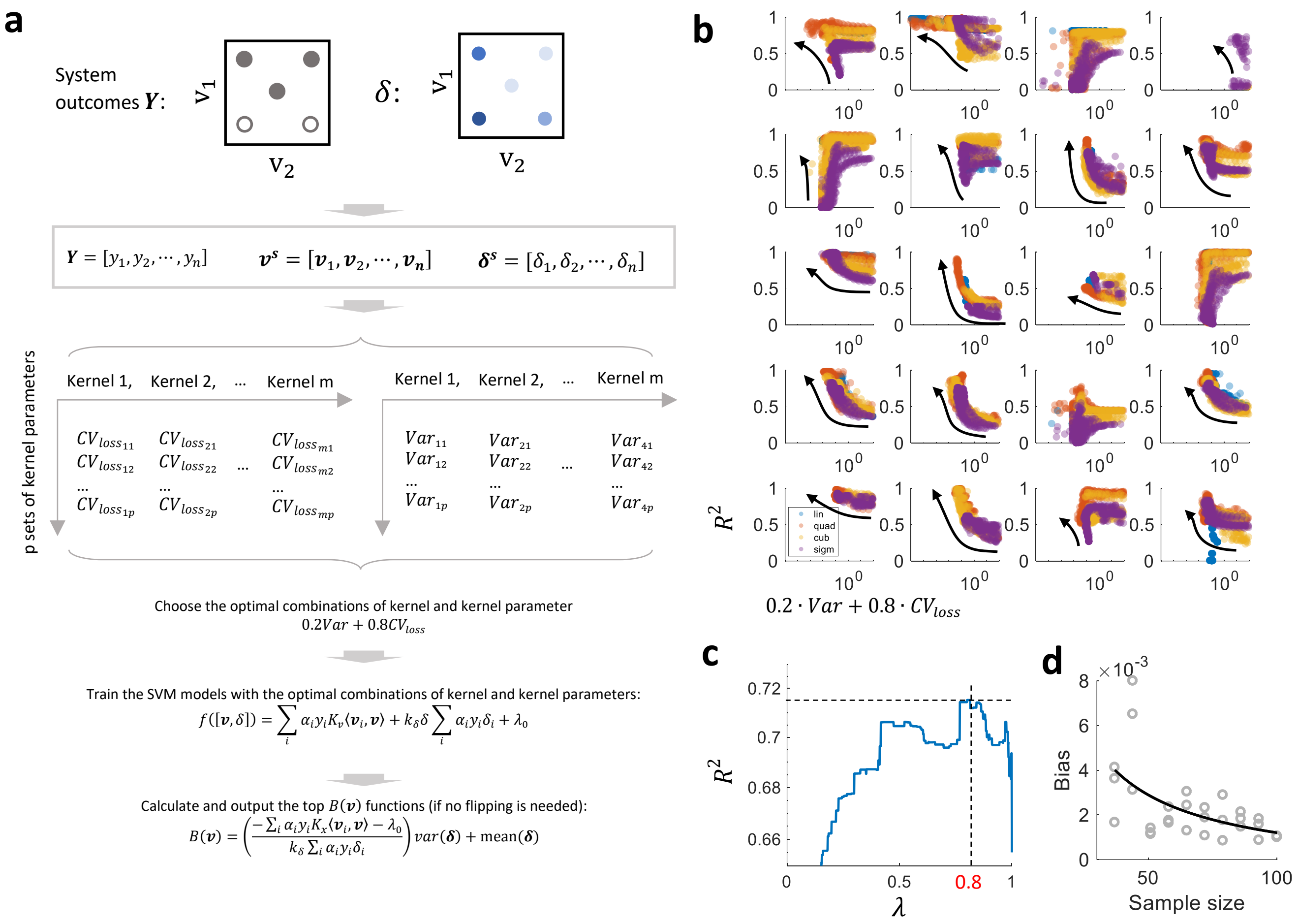


**Supplementary Figure 4: The general calibration procedure using SVM.**

**a.** General procedure of the calibration. $\boldsymbol{v}$ and $\delta$ are standardized to have mean of 0 and standard deviation of 1. The standardized data are used as inputs to train SVM models with different kernels and kernel parameters. Cross validation accuracy loss ($CV_{loss}$) and bootstrapped variance ($Var$) for each parameter combination are calculated to choose the optimal $B(\boldsymbol{v})$. Finally, calculate the top $B(\boldsymbol{v})$. See Supplementary Note 5.7 for a more detailed step-by-step graphical procedure.

**b.** To show that $0.2 \cdot Var + 0.8 \cdot CV_{loss}$ is a consistent indicator of high correlation (indicated by $R^2$ values) between $B(\boldsymbol{v})$ and the true $B$, we constructed 20 models that have known $B(\boldsymbol{v})$ (each subplot represents one model). Each dot represents the result of one set of kernel. These models show that minimizing $0.2 \cdot Var + 0.8 \cdot CV_{loss}$ leads to higher $R^2$ values (indicated by the black arrows), with a few exceptions. See Supplementary Note 5.5 for model and model parameters used in this panel.

**c.** Mean $R^2$ value at each $\lambda$ was calculated using the top 3 $B(\boldsymbol{v})$ selected by $(1-\lambda) \cdot Var + \lambda \cdot CV_{loss}$. The max mean $R^2$ occurs at $\lambda = 0.8$.

**d.** Bias decreases exponentially with increasing sample size. Using the model corresponding to the third subplot in the first column of panel b, we sampled a subset of the 100 data points without replacement to train $B(\boldsymbol{v})$ and the bias of $B(\boldsymbol{v})$ is calculated. The black trace represents the data points fitted to the function $Bias = a \cdot (Sample\ size)^{-\alpha} + b$.

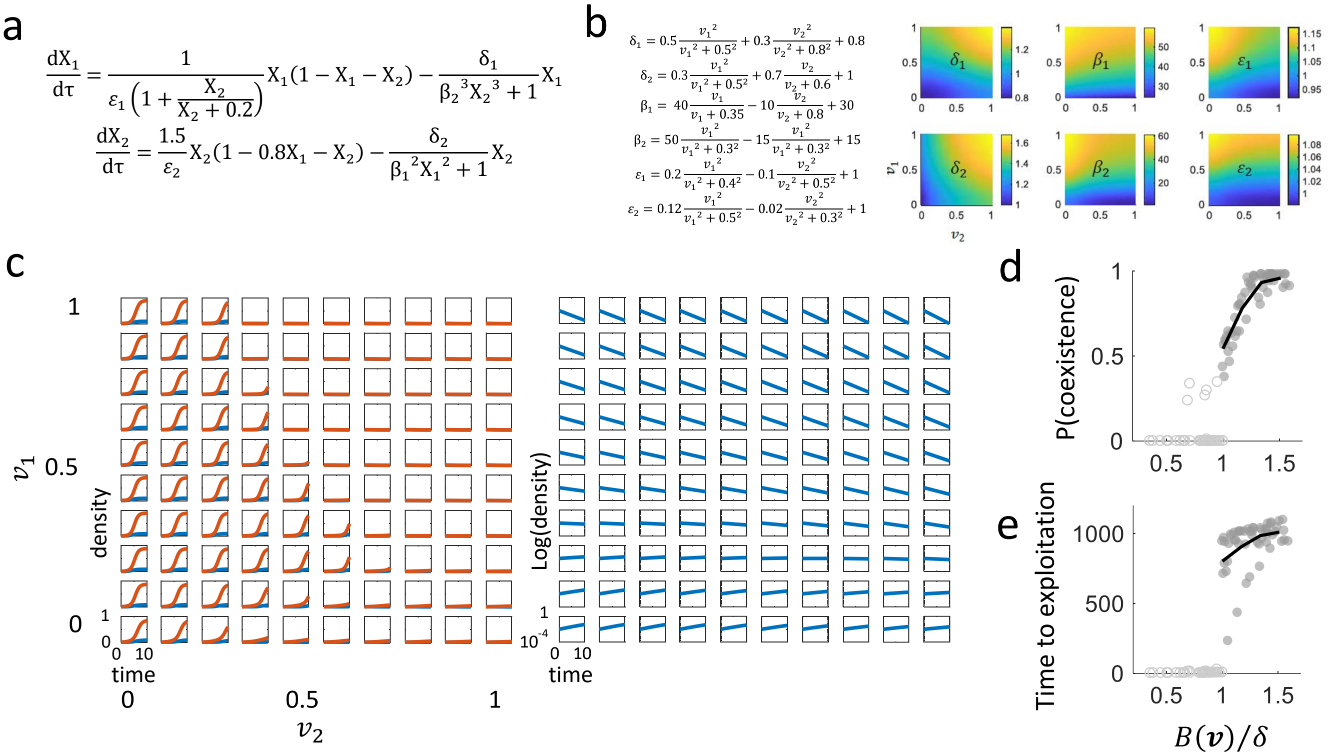


**Supplementary Figure 5: Detailed simulation and calibration procedure using a complex model.**

**a.** The model equations of a complex mutualistic system, which cannot be easily solved analytically.

**b.** Model parameters as functions of system variables $v_1$ and $v_2$. For an experimental system, $v_1$ and $v_2$ would correspond to experimentally controllable parameters, which could affect multiple mechanistic parameters simultaneously.

**c.** Simulated time courses with different $v_1$ and $v_2$ values. The group on the left simulates cocultures (blue: $X_1$ and red: $X_2$). The group on the right simulates monoculture, where $X_2 = 0$. The growth rates of the monoculture can then be used to calculate $\delta$. Note that we directly used the parameter value of $\delta$ in our procedure.

**d.** The calibrated $B(\boldsymbol{v})$ along with $\delta$ is predictive of probability of coexistence. To get the probability of coexistence at each $(v_1, v_2)$, we ran 100 simulations with 100 different ratios of initial densities while keeping the total initial density the same.

**e.** $B(\boldsymbol{v})/\delta$ is also predictive of how well the system can resist cheater exploitation. The y axis indicates the time the system can persist before the cheater populations compete out the cooperators.

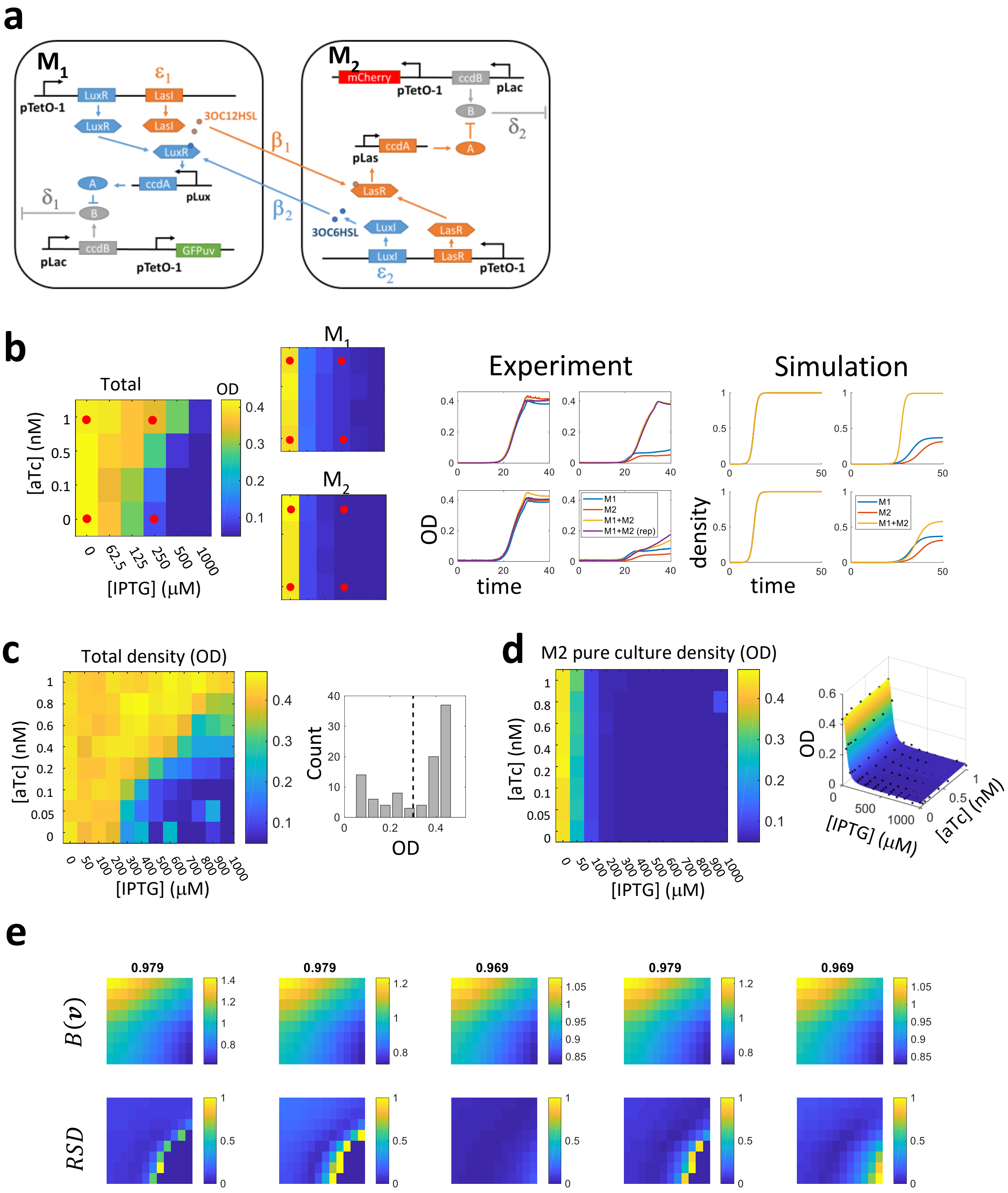


**Supplementary Figure 6: The QS-based mutualism system.**

**a.** The schematic of the synthetic gene circuit. $\delta_1$, $\delta_2$, $\beta_1$, $\beta_2$, $\varepsilon_1$, and $\varepsilon_2$ are indicated in the diagram to show the molecular mechanisms each term is primarily associated with.

**b.** Verification of the basic system dynamics. The final OD values of coculture and monocultures are recorded. Monocultures of $M_1$ and $M_2$ are significantly suppressed by IPTG, while the cocultures

exhibit synergistic growth. The total density of the cocultures increases with increasing [aTc]. The comparison between experimental and simulated temporal dynamics indicate that our model captures the basic mutualistic dynamics observed empirically. The four red dots in the heatmaps indicate the four experimental conditions of the simulated and experimental time courses. We used Supplementary Equations 3.22 and 3.23 in the Supplementary Information for the simulation. The parameter values are: $\delta_1([\text{IPTG}] = 0) = \delta_2([\text{IPTG}] = 0) = 0$ ; $\delta_1([\text{IPTG}] = 1000\mu\text{M}) = 0.63$ ; $\delta_2([\text{IPTG}] = 1000\mu\text{M}) = 0.68$ ; $\beta_1([\text{aTc}] = 0\mu\text{M}) = \beta_2([\text{aTc}] = 0\mu\text{M}) = 2$ ; $\beta_1([\text{aTc}] = 1\text{nM}) = \beta_2([\text{aTc}] = 1\text{nM}) = 200$; $\varepsilon_1 = \varepsilon_2 = 1$; $\rho = 1$.

**c.** Using higher resolution of [aTc] and [IPTG] gradients, we obtained the total density of cocultures using OD measurements. At 32 hours, a bimodal distribution of final densities emerges with a trough at OD=0.3.

**d.** Quantification of $\delta$ based on OD of $M_2$ monoculture at 32 hours. The final OD is fitted using $OD([aTc],[IPTG]) = V_a \frac{[aTc]^{n_a}}{[aTc]^{n_a}+k^{n_a}} + V_I \frac{k^{n_I}}{[IPTG]^{n_I}+k^{n_I}} + b$ to reduce noise of the resulting $\delta$ measurements. $\delta$ is then calculated by $\delta = 1 - \frac{OD([aTc],[IPTG])}{\max(OD([aTc],[IPTG]))}$.

**e.** The top 5 $B(\boldsymbol{v})$ and their corresponding relative standard deviation ($RSD$) (the axes are the same as heatmaps in panel c and d).

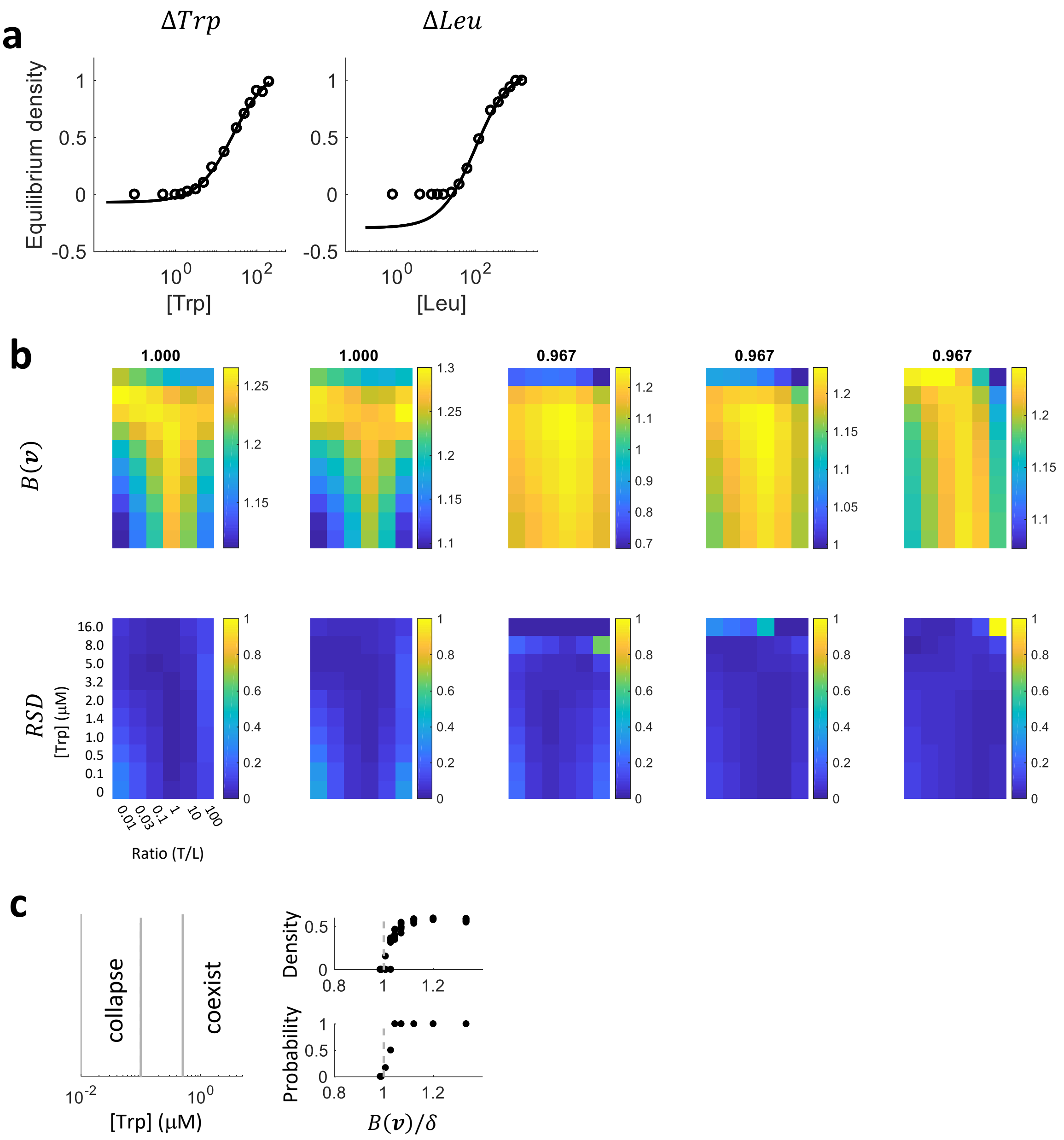


**Supplementary Figure 7: The yeast auxotroph system.**

**a.** The normalized equilibrium density of $\Delta Trp$ and $\Delta Leu$ were used to quantify $\delta$ for each strain. We only fitted density that is above the detection limit (10,000 cells/well). Since $\Delta Leu$ reaches below detection limit when [Leu] is relatively low, extrapolation is done to estimate the death rate at lower [Leu] (see Supplementary Figure 3a for the reasoning).

**b.** 5 top $B(\boldsymbol{v})$ show both consistency and discrepancy. All $B(\boldsymbol{v})$ indicate an optimal initial density at an intermediate level. However, when [Trp] increase to 16μM, $B(\boldsymbol{v})$ can either increase or decrease.

**c.** Our approach can also predict probability of coexistence. We excluded the ratio of initial density, leaving [Trp] to construct a one dimensional $\boldsymbol{v}$**.** The boundary between coexistence and collapse is between 0.1 μM tryptophan, 0.8 μM leucine and 0.5μM tryptophan, 4.0 μM leucine. To calibrate a $B(\boldsymbol{v})$ for this system, we assume supplementing the two amino acids does not change the cooperation capability. This calibration demonstrates that our procedure can also predict probability of coexistence for experimental systems.

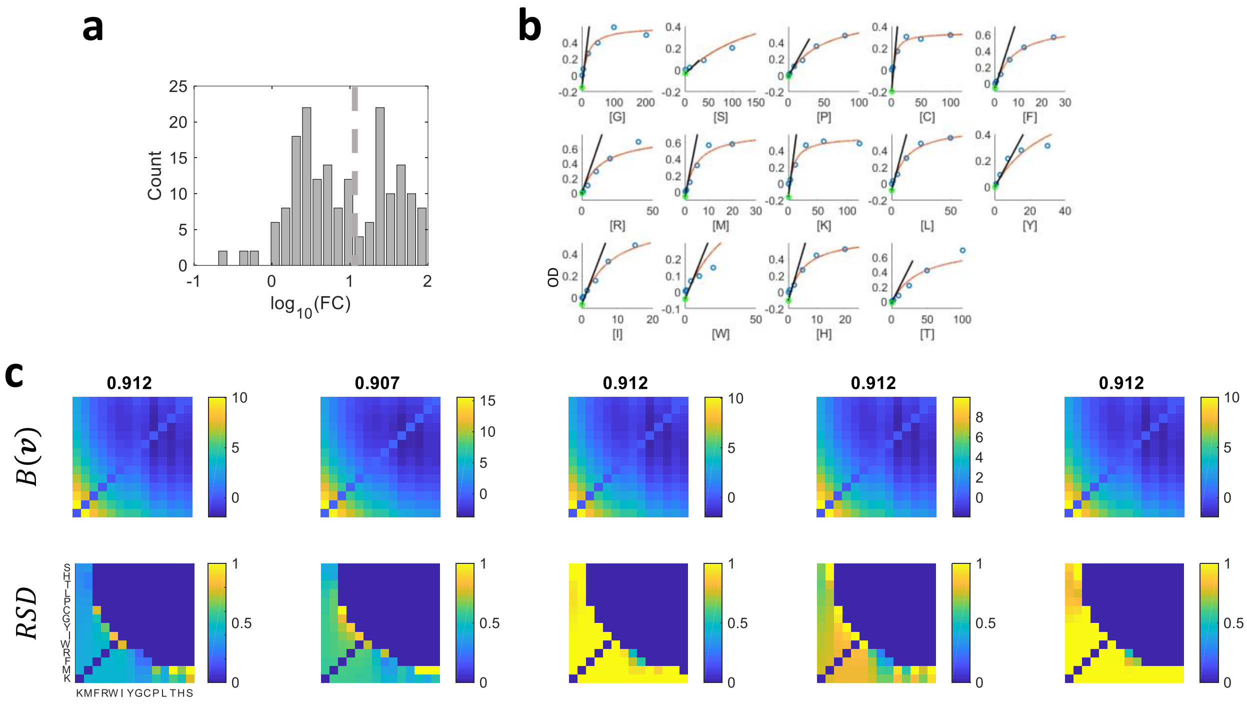


**Supplementary Figure 8: The 92 pairwise *E. coli* mutualistic systems constructed using 14 auxotrophic strains.**

**a.** The logarithm of fold change (FC) of total final density for the 92 systems exhibit a bimodal distribution. The trough of the bimodal distribution corresponds to FC=10 (dashed grey line), which is used to classify coexistence and collapse.

**b.** Quantification of $\delta$ for all 14 auxotrophs by fitting yield of the strain at different concentrations of their corresponding amino acid. Since none of the auxotrophs can grow without supplementary amino acid, $\delta \geq 1$ is expect for all auxotrophs at [AA]=0 (AA denotes the corresponding amino acid). Therefore, according to the reasoning demonstrated in Supplementary Figure 3a, extrapolation of the data is required. We only fitted the data points that have OD>0.1, since OD<0.1 can be below the linear detection range of microplate readers. The data are fitted and extrapolated with Hill equations (red curves) or using a linear extrapolation of the fitted curves (the black trace). Green circles represent the values of OD of the fitted Hill equations at [AA]=0. $\delta$ is then calculated by $1 - \frac{\mathrm{OD([AA]=0)}}{\mathrm{max(OD([AA]))}}$ for all 14 strains using $\mathrm{OD([AA]} = 0)$ obtained from either Hill equation or linear function. The two methods yield comparable results. We chose the $\delta$ calculated using the Hill equation for the calibration process.

**c.** The top calibrated $B(\boldsymbol{v})$ and their variability are indicated by relative standard deviation ($RSD$).

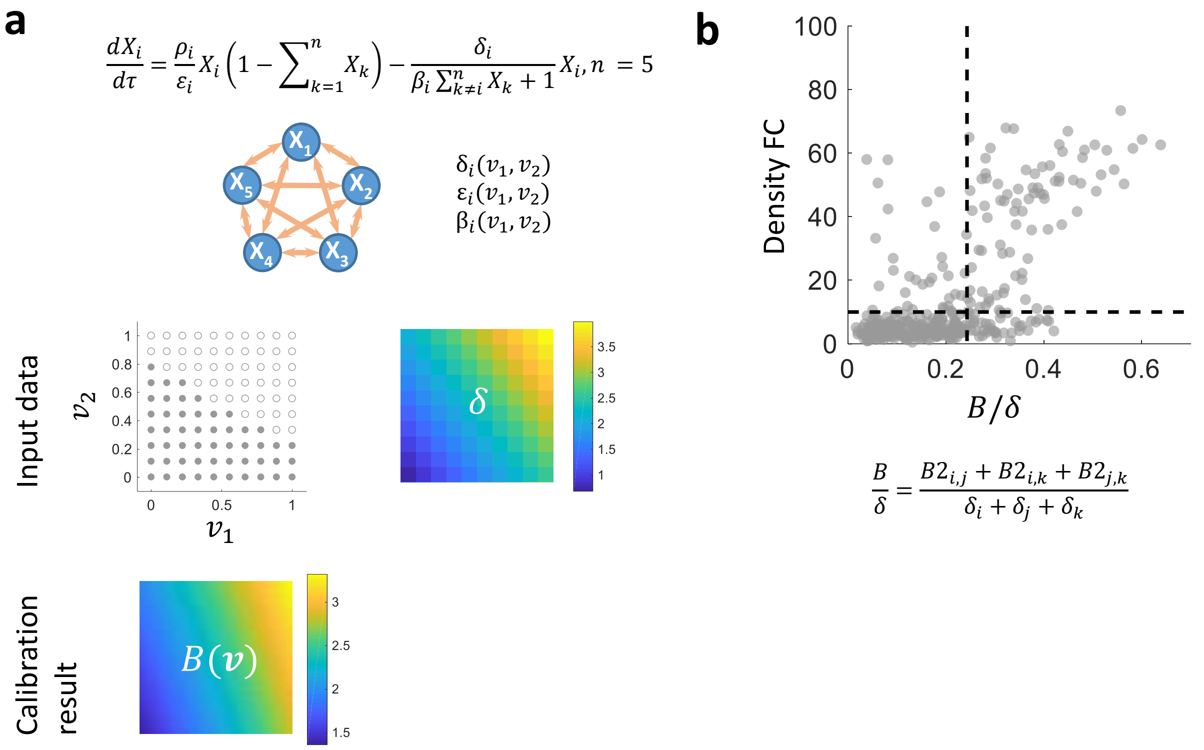


**Supplementary Figure 9: Mutualistic systems comprised of more than two partners.**

**a.** A mutualistic model with 5 partners. All the parameters in the model are linear functions of $v_1$ and $v_2$ and the parameters of the linear function are randomly generated.

**b.** Constructing a predictive metric for 3-member systems from calibrated results of 2-member systems. We first normalized the range of calibrated $B$ of 2-member systems ($B2$) to [0, 1]. The effective benefit of 3-member system is simply the average of $B2$ for all underlying 2-member systems and stress is the average of $\delta$ for all 3 members. The subscripts of $B2$ and $\delta$ represent the indices of the 3 members. We swept a threshold for $B/\delta$ between 0 and 1 to classify coexistence (FC≥10) versus collapse (FC<10). The prediction accuracy reaches the maximum of 80.8% at a threshold equals to 0.24.

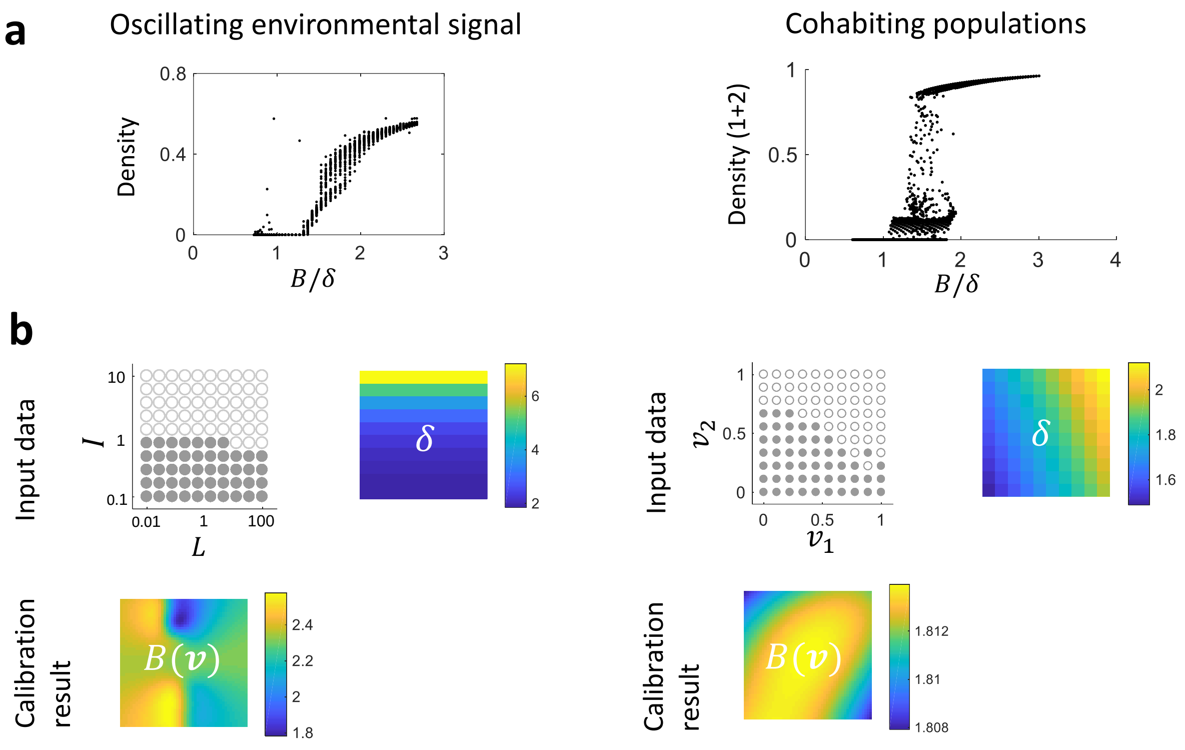


**Supplementary Figure 10: Mutualistic systems in dynamic environments.**

**a.** Two systems were analyzed: one system is a pairwise mutualism system that is modulated by oscillatory signals (the left panel) and the other system contains a pair of mutualists and 5 bystander populations that interact with the mutualists (the right panel). We verified that theoretically calculated $B/\delta$ roughly holds as a predictor for total density. The transition between coexistence and collapse, however, do not occur strictly at 1. Each dot represents a simulation result from one set of model parameters.

**b.** The input data used for the calibrations and the calibrated $B(\boldsymbol{v})$. The two $B(\boldsymbol{v})$ landscapes are used to generate the prediction plots shown in Figure 4c, d.

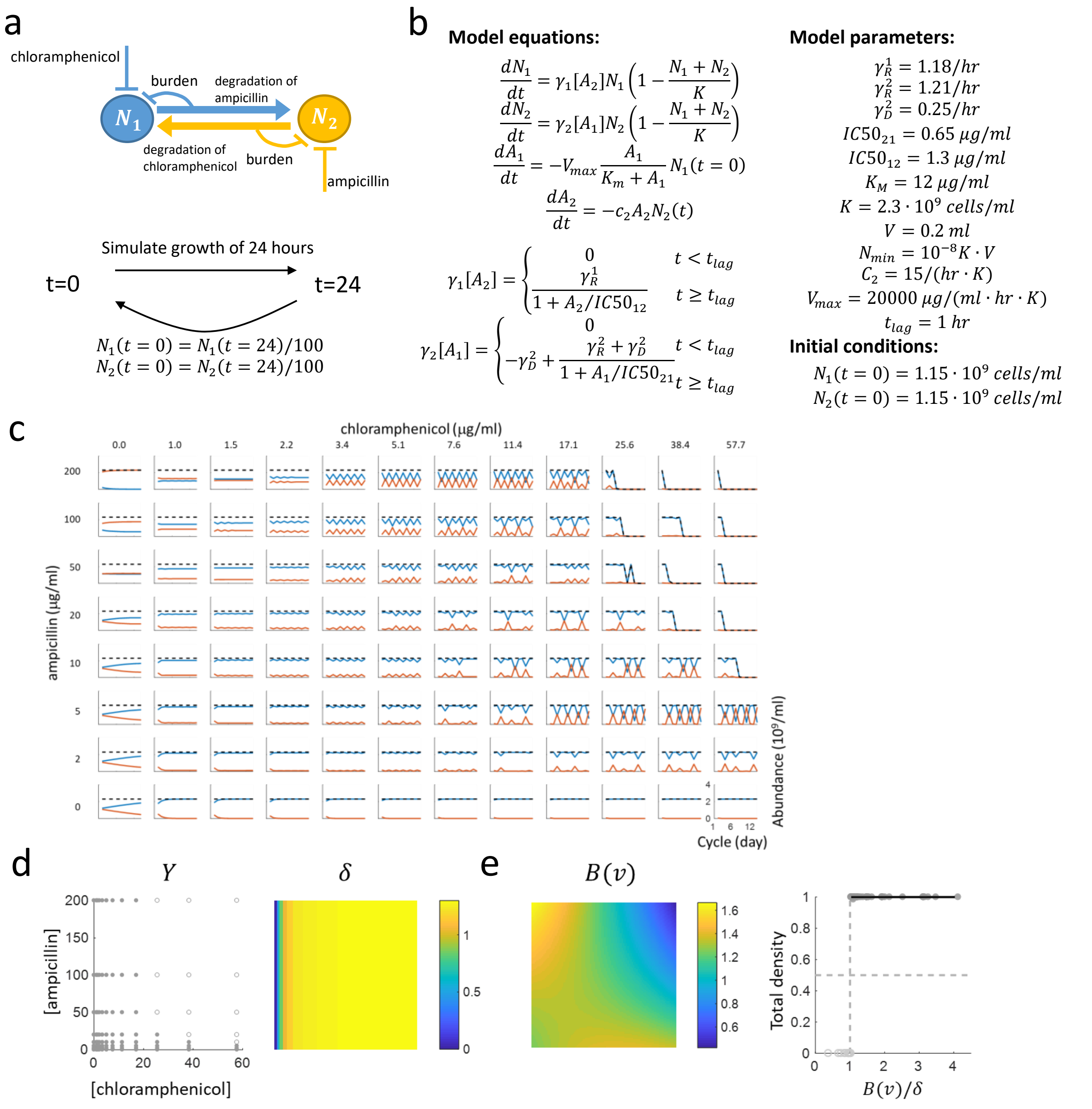


**Supplementary Figure 11: Application to a mutualistic system displaying oscillatory dynamics.**

a. System schematic and simulation procedure. $N_1$ is resistant to ampicillin and chloramphenicol imposes stress on $N_1$. $N_2$ is resistant to chloramphenicol and ampicillin imposes stress on $N_2$. Both strains can deactivate antibiotics that they are resistant to. The simulations are done by looping through 12 cycles where the initial density of $N_1$ and $N_2$ are set to be 1/100 of the final densities of the previous cycle.

**b.** Model equations are replicated from eq. [3] in SI of the original publication. Parameter values are copied from Figure S7 in the original publication.

**c.** Replication of time course in Figure S7 of the original publication. The model used in the original publication also incorporates molecular details of the interaction mechanisms. Red traces represent the

ampicillin resistant population and blue traces represent the chloramphenicol resistant population. Black dash lines represent total cell densities.

**d.** Input data of our calibration procedure. $\delta$ is estimated according to Figure 1D in the original publication.

**e.** Calibration results show that our calibration procedure still provides high prediction accuracy. The total densities corresponding to coexistence do not have a wide distribution.

# Supplementary Notes


1. Previous mutualism models ........ 21
   1. The Lotka-Volterra model ........ 21
   2. Other variants ........ 21
2. Building various mutualism models ........ 22
   1. Incorporation of stress ........ 22
   2. Model assumptions ........ 23
   3. Modifications of the logistic growth equation ........ 24
   4. Specific implementations of benefit, stress and cost ........ 25
   5. Examples of combined fitness impact of $\beta$ and $\varepsilon$ ........ 26
3. Derivation of diverse criteria ........ 27
   1. An example ........ 28
   2. Summary of criteria derived from symmetric models ........ 29
   3. Criteria from models with additional complexities ........ 29
4. Theoretical generality of the simple metric ........ 34
   1. Establish the general predictive power of $B/\delta$ ........ 34
   2. The predictive accuracy is maintained in the presence of noise ........ 35
5. Calibration using SVM ........ 36
   1. Kernels ........ 36
   2. Standardizing input data ........ 36
   3. From SVM output to calibrated $\boldsymbol{B}(\boldsymbol{v})$ ........ 36
   4. Cross validation and bootstrapping ........ 37
   5. Twenty sets of simulations to establish and test the calibration procedure ........ 37
   6. Calibration with simulated data with unknown $B(\boldsymbol{\theta})$: an example ........ 39
   7. Detailed graphical representation of the calibration procedure ........ 40
6. Framework generality verified by complex mutualistic systems ........ 42
   1. A 5-mutualist system ........ 42
   2. The experimental 3-member mutualistic systems ........ 42
   3. A mutualistic system in an oscillatory environment ........ 42
   4. A mutualistic system cohabiting with other populations ........ 43

# 1. Previous mutualism models

In this section, we briefly summarize some limitations of previously published models in studying the transition between mutualism coexistence and collapse.

## 1. The Lotka-Volterra model

Consider the following model that follows the basic form of Lotka-Volterra model of mutualism. This formulation is a nondimensionalized form of previous models[1-6]. The parameters are renamed according to our parameter assignments.

$$\frac{dX_1}{d\tau} = X_1(1 - \delta_1 X_1) + \beta_1 X_2 X_1 \quad (1.1)$$

$$\frac{dX_2}{d\tau} = \rho X_2(1 - \delta_2 X_2) + \beta_2 X_1 X_2 \quad (1.2)$$

Although the model formulation captures the logic of mutualism, it can generate unbounded growth of the two partners, which is not a biologically relevant state. For example, when $\rho = 1$, $\beta_1 = \beta_2 = 2$ and $\delta_1 = \delta_2 = 1$, the two populations are not bounded by their carrying capacities, but both grow exponentially. Importantly, the model does not capture population collapse so it cannot explain the transition between collapse and coexistence. Thus, this model formulation is not suitable for our purpose.

A coexistence criterion for mutualism can be derived using L-V model formulation and is previously demonstrated[1-5]:

$$\beta_1 \beta_2 < \rho \delta_1 \delta_2 \quad (1.3)$$

However, this criterion captures the transition between stable coexistence and unbounded growth. It also suggests that mutualism is destabilizing, since increasing the strength of mutualistic interaction ($\beta_1 \beta_2$) tends to violate the above condition. The interpretation of this criterion can be contradictory to empirical observations that mutualism stabilizes community[7-9]. Although Lotka-Volterra models are sufficient to answer many questions related to mutualistic systems, it has been proposed that this discrepancy between model dynamics and empirical observations can be attributed to its unrealistic assumptions[10-13].

## 2. Other variants

We found that general mutualism models that generate unbounded growth usually do not capture the transition between coexistence and collapse. For example, the following three models were established previously to find structurally stable mutualistic models[10]. In contrast to the L-V model which implements the interaction as a linear term, the following models present three alternative ways of adding the interaction to the basic logistic growth equation.

$$\begin{aligned} \frac{dN_1}{dt} &= r_1 N_1 \frac{K_1 - N_1 + \alpha_{12} N_2}{K_1 + \alpha_{12} N_2} \\ \frac{dN_2}{dt} &= r_2 N_2 \frac{K_2 - N_2 + \alpha_{21} N_1}{K_2 + \alpha_{21} N_1} \end{aligned} \quad (1.4)$$

$$\frac{\mathrm{d}N_1}{\mathrm{dt}} = r_1 N_1 \frac{K_1 - N_1 + \alpha_{12} N_2}{K_1}$$
$$\frac{\mathrm{d}N_2}{\mathrm{dt}} = r_2 N_2 \frac{K_2 - N_2 + \alpha_{21} N_1}{K_2} \qquad (1.5)$$

$$\frac{\mathrm{d}N_1}{\mathrm{dt}} = r_1 N_1 \frac{\left(1 + \frac{\alpha_{12} N_2}{K_1}\right)(K_1 - N_1)}{K_1}$$
$$\frac{\mathrm{d}N_2}{\mathrm{dt}} = r_2 N_2 \frac{\left(1 + \frac{\alpha_{21} N_1}{K_2}\right)(K_2 - N_2)}{K_2} \qquad (1.6)$$

In all these three cases, some parameter combinations generate unbounded growth. For example, $r_1 = r_2 = 1;\ K_1 = K_2 = 1;\ \alpha_{12} = \alpha_{21} = 2;\ N_{10} = N_{20} = 0.1$ generates unbounded growth for 1.4 and 1.5 and $r_1 = r_2 = -1;\ K_1 = K_2 = 1;\ \alpha_{12} = \alpha_{21} = 2;\ N_{10} = N_{20} = 2$ generates unbounded growth for 1.6. Unbounded growth has been recognized as a limitation of many mutualism models[14-16].

To avoid generating unbounded growth, one strategy is to introduce saturating benefit[5]. However, although preventing unbounded growth, these models may still not generate negative growth which can be potentially countered by the increase of partner density. For example, the following model is adapted from a previous work[13] which falls into this category. In addition, if no decreasing density is captured, this model will stabilize at its coexistence state with any positive initial density for both populations.

$$\frac{dN_1}{dt} = r_1 N_1 \left(1 - \delta_1 N_1 + \frac{\beta_{12} N_2}{1/\beta_2 + N_2}\right)$$
$$\frac{dN_2}{dt} = r_2 N_2 \left(1 - \delta_2 N_2 + \frac{\beta_{21} N_1}{1/\beta_1 + N_1}\right) \qquad (1.7)$$

Due to the limitations of previous mutualism models in studying the transition between coexistence and collapse, a more systematic way of modeling mutualism is required to both ensure the basic logic of mutualism and capture the transition between stable coexistence and collapse.

# 2. Building various mutualism models

The diversity of mutualistic systems impedes the formulation of a general mutualism model and the general proof of a single criterion. We generated various mutualism models to reflect the diversity of mutualistic systems in nature and examine how diverse the coexistence criteria are. We also aim to investigate whether there exists an invariant form that is preserved regardless of specific model implementations. If such an invariant form exists, it will then reveal a fundamental coexistence criterion that is originated from the basic logic of mutualism.

## 1. Incorporation of stress

We explicitly define stress as a reduction of growth rate or productivity of biological systems, which is consistent with previous works[17-20]. Stress is universal in biology because it is present whenever growth

rate is lower than the optimal growth rate. In mutualistic systems, many studies have shown that stress factors alter the basal fitness of individual mutualists. These factors include nutrient limitation[21], rising temperature[22,23], rising $CO_2$ levels[24], invasive species[25], etc. Beyond benefit and cost, it is known that these stress factors also determine mutualistic outcomes[26].

To study quantitatively how stress affects mutualistic outcomes, we include stress as a model parameter that reduces the growth rate or carrying capacity of individual mutualists in various biotic/abiotic contexts. In previous models, stress has been included as linear turn-over rate[21,27-30] or intraspecific competition[1,2]. Although these studies have more specific terms describing the downward pressure they capture, we use "stress" as an appropriate umbrella term.

Stress, thus defined, plays an essential qualitative role in mutualism. First, an absence of stress would mean that populations operate at maximum fitness. Such populations could not benefit from mutualism, because they would already be operating at their optimal level. Second, in mutualistic models the downward pressure can resolve the unrealistic exponential growth of mutualistic partners by imposing an upper limit to mutual benefit[10]. Third, using stress can dissect the baseline fitness of individual mutualists caused by biotic/abiotic factors from the effect of mutualistic interaction on fitness.

## 2. Model assumptions

The key step of generating various mutualism models is to establish the set of assumptions the models should follow. We first start off with the most apparent aspects of mutualism: benefit and cost. These two aspects lead to two assumptions:

a) Benefit shall *increase* growth rate or carrying capacity and is positively dependent on partner density.
b) Cost shall *decrease* growth rate or carrying capacity.

To study the transition between coexistence and collapse in mutualism systems, the models must be able to simulate collapse, leading to the third assumption where we explicitly introduce stress to achieve negative growth. In addition to generating negative growth, stress has a physical meaning which is the difference between maximal fitness and baseline fitness in the absence of the partner:

c) Stress shall produce negative growth of populations with some parameter combinations.

The fourth assumption follows assumption c) to reinforce the effects of benefit and cost in mutualistic interaction:

d) Negative growth of a population shall be potentially counteracted by benefit provided by a partner but is further strengthened by cost.

Even when all the above assumptions are satisfied, we still need to verify that a model does not generate unbounded growth with *any* parameter set.

## 3. Modifications of the logistic growth equation

After establishing a minimal set of assumptions, we then need to establish a systematic way of generating diverse models that satisfy these assumptions.

Mathematically, there are infinite possible implementations of a mutualistic system. We attempt to cover the different *common and plausible* forms of kinetic models that have been adopted in previous studies. Previous models have captured benefit, cost and stress in many ways. The following is a short summary of how benefit, stress and cost are modeled previously that serve as building blocks for our own models. We used Hill equations to capture saturating benefit, which is the same approach used in previous studies[13,21,27,31,32]. Cost is also an aspect that is widely modeled, which can be implemented many ways[13,33], such as independent or dependent of partner density. Although stress is not often generally discussed, one possible form of stress is self-regulation, also called density dependence or inter-population competition, which often appears in large-scale models[1-3]. Linear death rate (often imposed by dilution) is also another common form of stress[21,27-30].

Inspired by these observations, we first examined different methods to modify a logistic growth equation.

Consider a basic logistic growth equation:

$$\frac{dN}{dt} = \mu N\left(1 - \frac{N}{N_m}\right) \tag{2.1}$$

This equation only has two parameters, growth rate $\mu$ and carrying capacity $N_m$. If we can derive a non-dimensionalized logistic growth equation, it can be rewritten as:

$$\frac{dX}{d\tau} = X(1 - X)\ \ (\tau = \mu t,\ X = N/N_m) \tag{2.2}$$

The followings are common modifications of the above equation:

1. The growth rate can be modified by multiplying the right-hand-side with a constant:
$$\frac{dX}{d\tau} = \alpha X(1 - X) \tag{2.3}$$
This equation modifies the growth rate from 1 to $\alpha$, where increasing $\alpha$ increases growth rate and leaves the carrying capacity the same. $\alpha$ can take any real number in this case.
2. The carrying capacity can also be modulated, leaving the growth rate unchanged:
$$\frac{dX}{d\tau} = X(1 - \alpha X) \tag{2.4}$$
The carrying capacity becomes $1/\alpha$ and $\alpha > 0$. This formulation does not generate negative growth.
3. The "1" in the logistic growth equation can also be modified:
$$\frac{dX}{d\tau} = X(\alpha - X) \tag{2.5}$$
It can be rewritten as the same form of logistic growth equation:
$$\frac{dX}{d\tau} = \alpha X\left(1 - \frac{X}{\alpha}\right) \tag{2.6}$$
In this case, both growth rate and carrying capacity are scaled by a factor of $\alpha$. $\alpha$ can take any real number because if the carrying capacity is negative, the growth rate will also be negative and thus the model will only generate bounded growth.

4. A first order term can be added to the equation:

$$\frac{dX}{d\tau} = X(1 - X) - \alpha X \tag{2.7}$$

Although this modification is equivalent to the previous one, it is worth noting since this form is commonly used to represent death rate or turnover rate.

The above analysis demonstrates that 1, 3, and 4 are robust modifications that provide consistent model modulations with any real value of $\alpha$ parameter. Following this analysis, we will then model stress, benefit and cost at these three locations in the logistic growth equation to capture the four assumptions presented above for mutualistic interactions.

## 4. Specific implementations of benefit, stress and cost

To capture the model assumptions of a), b) and c), we used the following formulations to modify simple logistic growth equations at location 1, 3 or 4 mentioned above to capture the logic of mutualistic interaction.

a) *Benefit shall be positively dependent on partner density.*

This assumption can be modeled at all three locations by

$$\frac{\beta' X_2}{X_2 + 1/\beta}\ (\beta > 0, \beta' > 0) \text{ or } \frac{-1}{\beta X_2 + 1}\ (\beta > 0), \tag{2.8}$$

where $X_2$ represents partner density (same as below) and $\beta$ and $\beta'$ are measures of strength of benefit. In both cases, the benefit function is saturating, which facilitates the stabilization of population densities[5]. The following are two specific examples of functions 2.8:

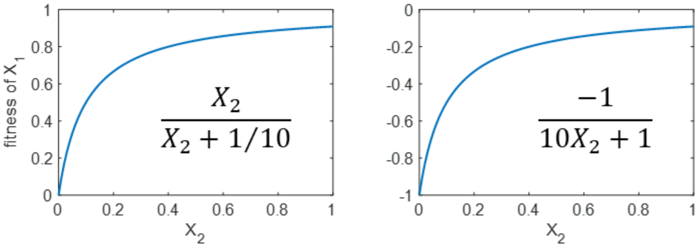


b) *Cost shall decrease growth rate or carrying capacity.*

We use $\varepsilon$ as a measure of level of cost. Specifically, this is implemented at location 1 or 3 by multiplication

$$\frac{1}{\varepsilon}(\varepsilon \geq 1) \text{ (density-independent) or } \frac{1}{\varepsilon X_2 + 1}\ (\varepsilon \geq 0) \text{ (density-dependent).} \tag{2.9}$$

Cost can also be implemented as a turnover rate at location 4

$$-\varepsilon\ (\varepsilon \geq 0) \text{ (density-independent) or } -\varepsilon X_2\ (\varepsilon \geq 0) \text{ (density-dependent).} \tag{2.10}$$

Two specific examples of the density-dependent functions in Supplementary Equations 2.9 and 2.10:

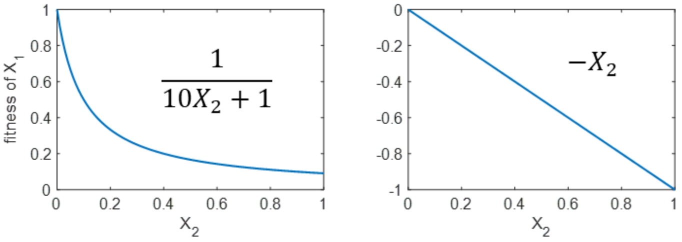

c) *Stress shall produce negative growth of populations with some parameter combinations.*
We use $\delta$ as a measure of stress. Specifically, this is implemented by modifying the logistic growth equation at location 1 or 3 by

$$1-\delta \text{ where } (\delta \geq 0) \tag{2.11}$$

Stress can also serve as a turnover rate at location 4

$$-\delta \tag{2.12}$$

Two or more of the parameters can share one location, so we permutated the three factors in the three locations and systematically generated 81 models (Supplementary Table 1). The 81 models are then checked against assumption d) and verify that the models do not generate unbounded growth. See Supplementary Software that investigates the model assumption d) and unbounded growth behavior. Out of the 81 models, 48 satisfy all 4 assumptions and do not generate unbounded growth with positive parameter values and positive initial densities.

## 5. Examples of combined fitness impact of $\boldsymbol{\beta}$ and $\boldsymbol{\varepsilon}$

Combining the two effects of $\beta$ and $\varepsilon$, we can examine different cases of the overall effect of $X_2$ density on $X_1$ fitness.

Model 25 in Supplementary Table 1 has $\beta$ modulating the growth rate and an effect of $\varepsilon$ independent of $X_2$:

$$25. \frac{dX_1}{d\tau} = \frac{\beta' X_2}{X_2 + 1/\beta} X_1(1 - X_1) - \delta\varepsilon X_1$$

Assuming $X_1 \ll 1$, the instant growth rate of $X_1$ becomes:

$$\frac{\beta' X_2}{X_2 + 1/\beta} - \delta\varepsilon \tag{2.13}$$

If we choose specific values for this function ($\beta' = 2$, $\beta = 10$, $\delta = 1$, $\varepsilon = 0.8$), the fitness of $X_1$ is:

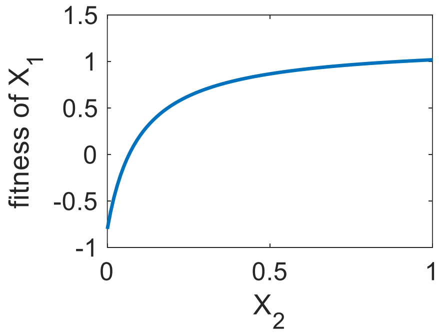


In this case, when the effect of $\varepsilon$ does not increase with partner density, fitness of $X_1$ is a monotonically increasing function of $X_2$.

As the next example, model 79 in Supplementary Table 1 only differs from model 25 in its density-dependent cost:

$$79. \frac{dX_1}{d\tau} = \frac{\beta' X_2}{X_2 + 1/\beta} X_1(1 - X_1) - \delta(1 + \varepsilon X_2) X_1$$

Assuming $X_1 \ll 1$, the instant growth rate of $X_1$ becomes:

$$\frac{\beta' X_2}{X_2 + 1/\beta} - \delta(1 + \varepsilon X_2) \tag{2.14}$$

If we use the same set of parameters as the previous example, we can see the overall fitness effect of both benefit and cost can increase at low partner density and decrease at high partner density:

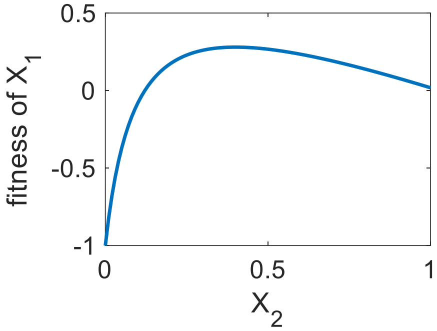


As the third example, another shape can be generated using model 73 in Supplementary Table 1:

$$73. \frac{dX_1}{d\tau} = \frac{1}{(1 + \varepsilon X_2)} \frac{\beta' X_2}{X_2 + 1/\beta} X_1(1 - X_1) - \delta X_1$$

where the instant growth rate of $X_1$ is:

$$\frac{1}{(1 + \varepsilon X_2)} \frac{\beta' X_2}{X_2 + 1/\beta} - \delta \tag{2.15}$$

Using $\beta' = 1$, $\beta = 100$, $\delta = 1$, $\varepsilon = 5$, we get the following relationship:

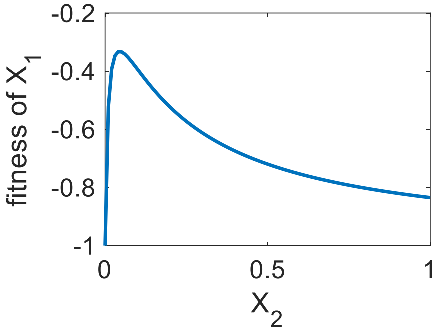


In this case, the overall effect of the interaction increases and decreases sharply at a low $X_2$ level (around 0.1 in this specific case).

With different function structures and parameter values, our model formulations capture several types of density vs. net effect curve as previously demonstrated[33], which include both monotonically increasing functions and biphasic dependencies.

# 3. Derivation of diverse criteria

This section demonstrates the diversity of criteria with various model formulations. We first use an example model to serve as the base model to demonstrate a detailed process to derive coexistence criteria. We then show the process of building various mutualism models with more complexity and deriving or approximating the corresponding criterion.

## 1. An example

Consider the following model as our base model for the analytical analyses:

$$\frac{dN_1}{dt} = \frac{\mu}{\varepsilon} N_1 \left(1 - \frac{N_1}{N_m}\right) - \frac{d\,K}{N_2 + K} N_1 \tag{3.1}$$

$$\frac{dN_2}{dt} = \frac{\mu}{\varepsilon} N_2 \left(1 - \frac{N_2}{N_m}\right) - \frac{d\,K}{N_1 + K} N_2 \tag{3.2}$$

The non-dimensionalized version of the model is (model 21 in Supplementary Table 1):

$$\frac{dX_1}{d\tau} = \frac{1}{\varepsilon} X_1(1 - X_1) - \frac{\delta}{\beta X_2 + 1} X_1 \tag{3.3}$$

$$\frac{dX_2}{d\tau} = \frac{1}{\varepsilon} X_2(1 - X_2) - \frac{\delta}{\beta X_1 + 1} X_2 \tag{3.4}$$

Where $\tau = \mu t, X_1 = X_2 = \frac{N}{N_m}, \delta = \frac{d}{\mu}, \beta = \frac{N_m}{K}$. The ranges of the parameters are: $\varepsilon \geq 1, \delta \geq 0, \beta \geq 0$

This system has 5 fixed points:

$$(0, 0), \tag{3.5}$$

$$(1 - \varepsilon\delta, 0), \tag{3.6}$$

$$(0, 1 - \varepsilon\delta), \tag{3.7}$$

$$\left(\frac{\beta - 1 + \sqrt{(\beta + 1)^2 - 4\beta\varepsilon\delta}}{4\beta}, \frac{\beta - 1 + \sqrt{(\beta + 1)^2 - 4\beta\varepsilon\delta}}{4\beta}\right), \tag{3.8}$$

$$\left(\frac{\beta - 1 - \sqrt{(\beta + 1)^2 - 4\beta\varepsilon\delta}}{4\beta}, \frac{\beta - 1 - \sqrt{(\beta + 1)^2 - 4\beta\varepsilon\delta}}{4\beta}\right), \tag{3.9}$$

By calculating the Jacobian, we found that the 4th fixed point (3.8) represents stable coexistence. The 5th fixed point is a saddle point and is unstable. For the 4th fixed point to be in the first quadrant of the Real domain, 2 conditions must be satisfied at the same time:

$$(\beta + 1)^2 - 4\beta\varepsilon\delta \geq 0 \tag{3.10}$$

$$\frac{\beta - 1 + \sqrt{(\beta + 1)^2 - 4\beta\varepsilon\delta}}{4\beta} > 0 \tag{3.11}$$

The first condition (Supplementary Equation 3.10) derives the coexistence criterion $\frac{(\beta+1)^2}{4\beta\varepsilon} \geq \delta$ and when this criterion is satisfied, the second condition is automatically satisfied if $\beta > 1$. When $\beta \leq 1$ the corresponding coexistence criterion is: $\frac{1}{\varepsilon} \geq \delta$. Since when $\beta \leq 1$, $\frac{(\beta+1)^2}{4\beta} \geq 1$. Thus, $\frac{(\beta+1)^2}{4\beta\varepsilon} \geq \frac{1}{\varepsilon} \geq \delta$ holds. Although the bound is not tight when $\beta \leq 1$, the overall criterion can still be described as $\frac{(\beta+1)^2}{4\beta\varepsilon} \geq \delta$ as a necessary condition. When $\beta \leq 1$, the model cannot capture obligatory mutualism, so we confine the range of $\beta$ to be $\beta > 1$ to ensure that both obligatory and facultative mutualism can be captured. Note that bifurcation analysis of the system shows Allee effect and how final density increases with increasing $\beta$, decreasing $\delta$ and decreasing $\varepsilon$.

## 2. Summary of criteria derived from symmetric models

Symmetric models in general generate interpretable analytical solutions. In Supplementary Table 1, all the models are assumed to be symmetric between two populations. We found that these symmetric models already generate a diverse set of coexistence criteria.

Most of these criteria are derived using the same logic used for the base model presented above, except for the criteria of model 1, 4, 28, 31, 55 and 58 in Supplementary Table 1, which are derived from setting the analytical solution of the saddle point lower than the initial density $X_0$. This is because the steady states that represent coexistence for these models are constants, whereas the saddle points are modulated by model parameters. For a detailed example of this derivation, refer to section 3.3.2.

In natural mutualism systems, cost can also scale with its partner's density[33]. In general, we observed that inclusion of density-dependent cost increases the complexity of the criteria. In addition, mutualists can also compete within the same niche[34]. In this case, the effective benefit term in general decreases from the model without competition, indicating the criteria also lumps the effect of competition.

## 3. Criteria from models with additional complexities

To relax some additional assumptions, such as complete symmetry, we also included additional levels of complexities to the base model by including asymmetry and turnover rate:

$$\frac{dN_1}{dt} = \frac{\mu_1}{\varepsilon_1} N_1 \left(1 - \frac{N_1 + \alpha\, N_2}{N_m}\right) - \frac{d_1\, K_2}{N_2 + K_2} N_1 - d_{01} N_1 \quad (3.12)$$

$$\frac{dN_2}{dt} = \frac{\mu_2}{\varepsilon_2} N_2 \left(1 - \frac{\alpha\, N_1 + N_2}{N_m}\right) - \frac{d_2 K_1}{N_1 + K_1} N_2 - d_{02} N_2 \quad (3.13)$$

After non-dimensionalization, the model becomes:

$$\frac{dX_1}{d\tau} = \frac{1}{\varepsilon_1}\ X_1(1 - X_1 - \alpha X_2) - \frac{\delta_1}{\beta_2 X_2 + 1} X_1 - \delta_{01} X_1 \quad (3.14)$$

$$\frac{dX_2}{d\tau} = \frac{1}{\varepsilon_2} \rho X_2(1 - \alpha X_1 - X_2) - \frac{\delta_2}{\beta_1 X_1 + 1} X_2 - \delta_{02} X_2 \quad (3.15)$$

where $\tau = \mu_1 t, X_1 = \frac{N_1}{N_m}, \rho = \frac{\mu_2}{\mu_1}, \delta_1 = \frac{d_1}{\mu_1}, \beta_1 = \frac{N_m}{K_1},\ X_2 = \frac{N_2}{N_m}, \delta_2 = \frac{d_2}{\mu_1}, \beta_2 = \frac{N_m}{K_2}$.

### *1. Criterion with two populations competing for resources*

Our base model assumes the two populations having separate carrying capacities. However, in natural settings, mutualists often share resources[15]. We can impose competition between them by adding $-\alpha X_2$ or $-\alpha X_1$ to location 3 in logistic growth equations of $X_1$ and $X_2$, respectively (number 82 in Supplementary Table 2):

$$\frac{dX_1}{d\tau} = \frac{1}{\varepsilon} X_1(1 - X_1 - \alpha X_2) - \frac{\delta}{\beta X_2 + 1} X_1 \quad (3.16)$$

$$\frac{dX_2}{d\tau} = \frac{1}{\varepsilon} X_2(1 - \alpha X_1 - X_2) - \frac{\delta}{\beta X_1 + 1} X_2 \quad (3.17)$$

Using the same method described in section 3.1, we derived the criterion of this model: $\frac{(\beta+\alpha+1)^2}{4\beta(\alpha+1)\varepsilon} > \delta$.

*2. Criterion considering initial density*

For this analysis, we used a symmetric model (model 83 in Supplementary Table 2):

$$\frac{dX_1}{d\tau} = \frac{1}{\varepsilon} X_1(1 - X_1) - \frac{\delta}{\beta X_2 + 1} \tag{3.18}$$

$$\frac{dX_2}{d\tau} = \frac{1}{\varepsilon} X_2(1 - X_2) - \frac{\delta}{\beta X_1 + 1} \tag{3.19}$$

Because most mutualism models generate Allee effect, coexistence can also be affected by initial density ($x_0$). However, we can derive a benefit term that depends on the initial density $x_0$ (for both $X_1$ and $X_2$) to predict deterministically whether the two populations coexist or not. To coexist, $x_0$ needs to be greater than the saddle point:

$$x_0 > \frac{\beta - 1 - \sqrt{(\beta + 1)^2 - 4\beta\varepsilon\delta}}{4\beta} \tag{3.20}$$

If we rearrange this inequality, we get

$$\frac{(1 - x_0)x_0\beta + (1 - x_0)}{\varepsilon} > \delta \tag{3.21}$$

*3. Criterion with asymmetric growth rate, cost, stress, and benefit*

Asymmetric parameters of the mutualism are more realistic in capturing real-world mutualism systems, so we relaxed the assumptions that cost ($\varepsilon$), rescue strength ($\beta$) and stress ($\delta$) are symmetric for both populations. The asymmetry of growth rate is captured by $\rho$. In the following model (model 84 in Supplementary Table 2), we assumed $X_1$ and $X_2$ share the same carrying capacity. We found that separated carrying capacity also yields similar results.

$$\frac{dX_1}{d\tau} = \frac{1}{\varepsilon_1} X_1(1 - X_1 - X_2) - \frac{\delta_1}{\beta_2 X_2 + 1} X_1 \tag{3.22}$$

$$\frac{dX_2}{d\tau} = \frac{1}{\varepsilon_2} \rho X_2(1 - X_1 - X_2) - \frac{\delta_2}{\beta_1 X_1 + 1} X_2 \tag{3.23}$$

This model has 5 fixed points:

$$(0, 0) \tag{3.24}$$

$$(1 - \varepsilon_1\delta_1, 0) \tag{3.25}$$

$$\left(0, 1 - \frac{\varepsilon_2\delta_2}{\rho}\right) \tag{3.26}$$

$$\left(\frac{\frac{\varepsilon_2\delta_2}{\rho}\sqrt{A} - \beta_2\varepsilon_2\delta_2 + \beta_1\varepsilon_2\delta_2 - 2\beta_1\varepsilon_1\delta_1\rho + \beta_1\beta_2\varepsilon_2\delta_2}{2\beta_1(\beta_1\varepsilon_1\delta_1\rho + \beta_2\varepsilon_2\delta_2)}, \frac{\varepsilon_1\delta_1\sqrt{A} - \beta_1\varepsilon_1\delta_1\rho + \beta_2\varepsilon_1\delta_1\rho - 2\beta_2\varepsilon_2\delta_2 + \beta_1\beta_2\varepsilon_1\delta_1\rho}{2\beta_2(\beta_1\varepsilon_1\delta_1\rho + \beta_2\varepsilon_2\delta_2)}\right) \tag{3.27}$$

$$\left(\frac{-\frac{\varepsilon_2\delta_2}{\rho}\sqrt{A} - \beta_2\varepsilon_2\delta_2 + \beta_1\varepsilon_2\delta_2 - 2\beta_1\varepsilon_1\delta_1\rho + \beta_1\beta_2\varepsilon_2\delta_2}{2\beta_1(\beta_1\varepsilon_1\delta_1\rho + \beta_2\varepsilon_2\delta_2)}, \frac{-\varepsilon_1\delta_1\sqrt{A} - \beta_1\varepsilon_1\delta_1\rho + \beta_2\varepsilon_1\delta_1\rho - 2\beta_2\varepsilon_2\delta_2 + \beta_1\beta_2\varepsilon_1\delta_1\rho}{2\beta_2(\beta_1\varepsilon_1\delta_1\rho + \beta_2\varepsilon_2\delta_2)}\right) \tag{3.28}$$

where $A = \rho^2(\beta_1\beta_2 + \beta_1 + \beta_2)^2 - 4\beta_1\beta_2\rho(\beta_1\varepsilon_1\delta_1\rho + \beta_2\varepsilon_2\delta_2)$.

Use the same logic presented in section 3.1, the following criterion is a necessary condition for the 4th fixed point to be present in the positive Real domain:

$$\frac{\rho(\beta_1\beta_2+\beta_1+\beta_2)^2}{4\beta_1\beta_2} \geq \beta_1\varepsilon_1\delta_1\rho+\beta_2\varepsilon_2\delta_2 \tag{3.29}$$

This criterion can also be rewritten as $\frac{\rho\left(\beta_1+\frac{1}{\theta_1}+1\right)^2}{4\beta_1\varepsilon_1\left(\frac{\rho}{\theta_1}+\theta_2\right)} \geq \delta_1$ where the asymmetry is captured by $\theta_1 = \frac{\beta_2}{\beta_1}$ and $\theta_2 = \frac{\varepsilon_2\delta_2}{\varepsilon_1\delta_1}$. Note that we specifically used $X_1$ as the reference population.

## 4. *Criterion with turnover rate and asymmetric growth rate*

If we consider both the asymmetry in growth rate ($\rho$) and a turnover rate $\delta_0$, the model becomes (model 85 in Supplementary Table 2):

$$\frac{dX_1}{d\tau} = \frac{1}{\varepsilon}\ X_1(1-X_1-X_2) - \frac{\delta}{\beta X_2+1}X_1 - \delta_0 X_1 \tag{3.30}$$

$$\frac{dX_2}{d\tau} = \frac{1}{\varepsilon}\rho X_2(1-X_1-X_2) - \frac{\delta}{\beta X_1+1}X_2 - \delta_0 X_2 \tag{3.31}$$

The analytical solution of this model is complex and involves solving 4th order polynomials. However, we can introduce the concept of correction terms to approximate the criterion. When we only add $\rho$ in the model and assume $\delta_0 = 0$, we get the following criterion:

$$\frac{(\beta+2)^2}{4\beta\varepsilon}\left(\frac{\rho}{\rho+1}\right) \geq \delta \tag{3.32}$$

In addition, when we only add the $\delta_0$ term in the model and assume $\rho = 1$, we get

$$\frac{(\beta(1-\varepsilon\delta_0)+2)^2}{4\beta\varepsilon} \geq \delta. \tag{3.33}$$

We hypothesized that the criterion with both correction terms $\left(\frac{\rho}{\rho+1}\right)$ and $(1-\varepsilon\delta_0)$ can approximate the criterion for model 3.30-31. The criterion for $\delta_0 > 0$ is:

$$\frac{(\beta(1-\varepsilon\delta_0)+2)^2}{4\beta\varepsilon}\left(\frac{\rho}{\rho+1}\right) \geq \delta \tag{3.34}$$

The accuracy of this criterion is evaluated by substitution of variables:

$$\begin{cases} z_1 = (1-X_1{}^*-X_2{}^*) - \delta_0\varepsilon \\ z_2 = X_2{}^* + \dfrac{1}{\beta} \end{cases} \tag{3.35}$$

where $X_1{}^*$ and $X_2{}^*$ represent fixed points of the model. $\frac{dX_1}{d\tau} = 0$ and $\frac{dX_2}{d\tau} = 0$ can then be written as

$$\begin{cases} z_2 = \dfrac{\delta\varepsilon}{\beta z_1} \\ \rho z_1 + (\rho-1)\delta_0\varepsilon + \dfrac{\delta\varepsilon}{\beta(z_1+z_2) - (\beta(1-\delta_0\varepsilon)+2)} = 0 \end{cases} \tag{3.36}$$

After substitution of $z_2$ with $z_1$ in the second equation, we get

$$\beta {z_1}^2 - (\beta(1-\delta_0\varepsilon)+2)z_1 + \frac{\rho+1}{\rho}\delta\varepsilon = \frac{\frac{\rho-1}{\rho}\delta_0\delta\varepsilon^2}{\rho z_1 + (\rho-1)\delta_0\varepsilon} \tag{3.37}$$

The left-hand side is a quadratic equation of has $z_1$, which has $\Delta = (\beta(1-\delta_0\varepsilon)+2)^2 - 4\frac{\rho+1}{\rho}\beta\delta\varepsilon$.

$$\Delta \geq 0 \Leftrightarrow \frac{(\beta(1-\varepsilon\delta_0)+2)^2}{4\beta\varepsilon}\left(\frac{\rho}{\rho+1}\right) \geq \delta \tag{3.38}$$

Thus, we know that the criterion is more accurate when $\frac{\frac{\rho-1}{\rho}\delta_0\delta\varepsilon^2}{\rho z_1+(\rho-1)\delta_0\varepsilon} \longrightarrow 0$. The approximated criterion is accurate when $\rho = 1$.When $\rho \to +\infty$ and $\delta_0 \to 0$, the criterion will also provide good approximations.

## *5. The criterion for N-mutualist systems*

The following model is used to determine the criterion with N-mutualist model:

$$\frac{dX_i}{d\tau} = \frac{1}{\varepsilon}X_i\left(1-\sum_{k=1}^{n}X_k\right) - \frac{\delta}{\beta\sum_{k\neq i}^{n}X_k+1}X_i \tag{3.39}$$

The non-trivial steady state can be solved by solving the following equation for X*:

$$\frac{n}{\varepsilon}X^*(1-nX^*) - \frac{n\delta}{\beta(n-1)X^*+1}X^* = 0 \tag{3.40}$$

Using the same strategy in section 3.1, we derive the criterion for coexistence:

$$\frac{(\beta+\frac{n}{n-1})^2}{4\beta\varepsilon\frac{n}{n-1}} > \delta \tag{3.41}$$

where benefit becomes a function of both $\beta$ and *n*. This result suggests that mutualism system can tolerate higher stress levels with increasing number of mutualists.

## *6. Mutualism model including cheater exploitation*

The mutualism model including cheaters is adapted from Supplementary Equations 3.22-23:

$$\frac{dX_1}{d\tau} = \frac{1}{\varepsilon}X_1(1-X_1-X_2-X_{1c}-X_{2c}) - \frac{\delta}{\beta X_2+1}X_1 - \varphi X_1 \tag{3.42}$$

$$\frac{dX_2}{d\tau} = \frac{1}{\varepsilon}X_2(1-X_1-X_2-X_{1c}-X_{2c}) - \frac{\delta}{\beta X_1+1}X_2 - \varphi X_2 \tag{3.43}$$

$$\frac{dX_{1c}}{d\tau} = X_{1c}(1-X_1-X_2-X_{1c}-X_{2c}) - \frac{\delta}{\beta X_2+1}X_{1c} + \varphi X_1 \tag{3.44}$$

$$\frac{dX_{2c}}{d\tau} = X_{2c}(1-X_1-X_2-X_{1c}-X_{2c}) - \frac{\delta}{\beta X_1+1}X_{2c} + \varphi X_2 \tag{3.45}$$

We assume 1) cheaters and cooperators share the same carrying capacity, 2) cheaters accept benefit produced by cooperators but do not provide benefit, thus do not experience cost, and 3) there is a constant

transfer rate ($\varphi$) from cooperator to cheater, representing mutation from the cooperator phenotype to cheater phenotype. Although this model does not generate stable coexistence of the cooperators, the time it takes for cheaters to take over the cooperators can serve as a metric for how stable the mutualistic system is.

### 7. *Model structures that generate lower boundary for stress*

We notice that to coexist, some model formulations not only require an upper boundary of $\delta$, but also require a lower boundary as well. If $\delta$ is lower than a threshold, the system can be dominated by the population that is more fit. However, this lower boundary occurs due to the system dynamic shifting from a mutualism-dominated mode to a competition-dominated mode.

There are two types of system dynamics that can lead to a loss of partner. One is due to high stress where the weaker partner will go extinct and the stronger partner will suffer from the loss of its partner. The other is due to lack of stress where the system shifts to a competition-dominated interaction where the fitter partner survives better by excluding its partner. In our study, we only focus on the first type of partner loss since the second type is a dynamic of competitive systems instead of mutualistic systems.

In general, we observed that a model needs to be both asymmetric and potentially competitive to create a scenario where the fitter population excludes the weaker population. The lower boundary for $\delta$ decreases when the weaker population gives out more benefit to its partner or reduction of competition. This observation can potentially explain why some mutualistic systems transition into competitive exclusion when external stress is reduced[21,35].

To demonstrate the relationships between these two transitions, we use the following model as an example, which is expressed in Supplementary Equations 3.30 and 30.31. The model incorporates both asymmetry and competition. Here we show two scenarios: the left panel includes competition ($a < 1$) and the right panel has no competition ($a = 1$). Using $\varepsilon = 1.2$, $\beta = 10$ and $\delta_0 = 0.1$, we generated the following phase diagrams of system behaviors.

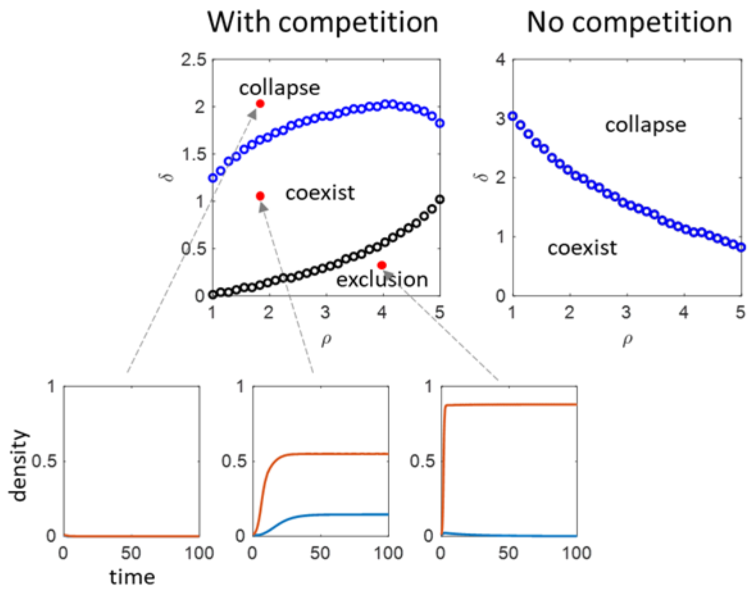

The blue boundaries represent the transition between coexistence and collapse, which is dictated by $B/\delta$. The black boundary represents transition from coexistence to competitive exclusion. The time courses show the decrease of fitness of both populations after transitioned into collapse from coexistence. On the other hand, the persisting population increases in density when transitions into competitive exclusion. The two transitions are two distinct behaviors. Both boundaries exist for $\delta$ when there is competition. However, the black boundary does not exist if the model does not incorporate competition, which demonstrates that transitioning to competitive exclusion is a model behavior generated by competition and cannot be generated by mutualism *alone*.

# 4. Theoretical generality of the simple metric

To establish the generality of the theoretical criterion, we verified that it is applicable to both symmetric and asymmetric mutualistic systems. We also want to test that the predictive power of the predictor is maintained when the partners are obligatory or facultative. Because mutualists can compete for the same resources in nature, we also verified that coexistence of partners that compete for resources also can be depicted by the criterion. We show some typical results of the predictive power of the criterion in these cases with Supplementary Figure 2.

## 1. Establish the general predictive power of $\boldsymbol{B/\delta}$

Figure 1f is generated using the model described by Supplementary Equations 3.16 and 3.17, where $\varepsilon \in [1, 1.2]$, $\delta \in [0, 2]$, $\beta \in [2, 5]$ and $a = 1$. The positive trend holds when the mutualism is facultative ($\delta < 1$).

Supplementary Figure 2a is also generated using the model presented by Supplementary Equations 3.16 and 3.17. Both are generated with $\beta \in [2, 10]$, $\delta \in [1.2, 2]$, $\varepsilon \in [1, 1.2]$ and $a = 1$. The panel on the left assumes $\mathrm{X}_1$ and $\mathrm{X}_2$ have the same initial density which is a uniformly distributed between 0 and 0.5. The panel on the right assumes changing of individual initial densities in a linear fashion while maintaining the sum of initial density of $\mathrm{X}_1$ and $\mathrm{X}_2$ at 1.

Supplementary Figure 2b is generated with the model presented above in Supplementary Equations 3.42-3.45, where $\varphi = 10^{-3}$, $\beta \in [5, 20]$ $\delta \in [1.2, 1.3]$, and $\varepsilon \in [1.2, 1.3]$. The initial densities are $\mathrm{X}_{10} = 0.1$, $\mathrm{X}_{10} = 0.1$, $\mathrm{X}_{1c} = 0$ and $\mathrm{X}_{2c} = 0$. The time to cheater exploitation is quantified by the first time point where the total density of cooperators drops below their initial total density due to overwhelming competitions from the cheater populations. Other time to cheater exploitation in this study is quantified using the same method.

Supplementary Figure 2c is generated by

$$\frac{dX_1}{d\tau} = \frac{1}{\varepsilon_1} X_1(1 - X_1) - \frac{\delta_1}{\beta_2 X_2 + 1} X_1 \tag{4.1}$$

$$\frac{dX_2}{d\tau} = \frac{1.2}{\varepsilon_2} X_2(1 - X_2) - \frac{\delta_2}{\beta_1 X_1 + 1} X_2 \tag{4.2}$$

Where the left panel uses parameter values of $\beta_1 = 2$, $\beta_2 \in [2, 5]$, $\delta_1 = 1.2$, $\delta_2 \in [0.5, 2]$, $\varepsilon_1 = 1.1$, and $\varepsilon_2 \in [1, 1.2]$. The initial densities are $X_{10} = 1$ and $X_{20} = 1$. The right panel uses parameter values of $\beta_1 = 2$, $\beta_2 \in [2, 5]$, $\delta_1 = 0.8$, $\delta_2 \in [1, 2]$, $\varepsilon_1 = 1.1$, and $\varepsilon_2 \in [1, 1.2]$. The initial densities are $X_{10} = 1$ and $X_{20} = 1$. This panel specifically tests the cases where the survival of the partners is fully dependent on each other.

Supplementary Figure 2d is generated by 3.22-3.23, where the left panel uses parameter values of $\rho = 1.2$, $\beta_1 = 2$, $\beta_2 \in [5, 10]$, $\delta_1 = 1.2$, $\delta_2 \in [0.5, 2]$, $\varepsilon_1 = 1.1$, and $\varepsilon_2 \in [1, 1.2]$. The initial densities are $X_{10} = 1$ and $X_{20} = 1$. The right panel uses parameter values of $\beta_1 = 2$, $\beta_2 \in [5, 10]$, $\delta_1 = 0.8$, $\delta_2 \in [1, 2]$, $\varepsilon_1 = 1.1$, and $\varepsilon_2 \in [1, 1.2]$. The initial densities are $X_{10} = 1$ and $X_{20} = 1$. This panel specifically tests the cases where one mutualist's survival is not fully dependent on the other.

Supplementary Figure 2e is generated using the same model as the above. Where the parameter values are $\beta_1 = 5$, $\beta_2 \in [2, 10]$, $\delta_1 = 1.2$, $\delta_2 \in [1.2, 2]$, $\varepsilon_1 = 1.1$, and $\varepsilon_2 \in [1, 1.2]$. The sum of initial densities is kept at 0.3 and the values of $\mathrm{X}_{10}$ and $\mathrm{X}_{20}$ are changed in a linear fashion. 1000 different combinations of parameters are used and 50 different $\mathrm{X}_{10}\!:\!\mathrm{X}_{20}$ simulations are performed with each parameter combination to calculate the probability of coexistence.

Supplementary Figure 2f is generated using the following model, which is based on the symmetric model presented in Supplementary Equations 3.42-45, while adding asymmetry:

$$\frac{dX_1}{d\tau} = \frac{1}{\varepsilon_1} X_1(1 - X_1 - X_2 - X_{1c} - X_{2c}) - \frac{\delta_1}{\beta_2 X_2 + 1} X_1 - \varphi X_1 \tag{4.3}$$

$$\frac{dX_2}{d\tau} = \frac{1.3}{\varepsilon_2} X_2(1 - X_1 - X_2 - X_{1c} - X_{2c}) - \frac{\delta_2}{\beta_1 X_1 + 1} X_2 - \varphi X_2 \tag{4.4}$$

$$\frac{dX_{1c}}{d\tau} = X_{1c}(1 - X_1 - X_2 - X_{1c} - X_{2c}) - \frac{\delta_1}{\beta_2 X_2 + 1} X_{1c} + \varphi X_1 \tag{4.5}$$

$$\frac{dX_{2c}}{d\tau} = 1.3 X_{2c}(1 - X_1 - X_2 - X_{1c} - X_{2c}) - \frac{\delta_2}{\beta_1 X_1 + 1} X_{2c} + \varphi X_2 \tag{4.6}$$

Where $\varphi = 10^{-3}$, $\beta_1 = 4$, $\beta_2 \in [40, 60]$, $\delta_1 = 1.8$, $\delta_2 \in [1.4, 1.5]$, $\varepsilon_1 = 1.4$, $\varepsilon_2 \in [1, 1.2]$. The initial densities are $\mathrm{X}_{10} = 0.05$, $\mathrm{X}_{10} = 0.05$, $\mathrm{X}_{1c} = 0$ and $\mathrm{X}_{2c} = 0$.

## 2. The predictive accuracy is maintained in the presence of noise

In addition to investigating the probability of coexistence when initial density is randomly distributed (Supplementary Figure 2a, 2e), we also explicitly modeled noise to test the effect of noise on the prediction accuracy of the criterion. We added Gaussian noise to our base model ($\eta$):

$$\frac{dX_1}{d\tau} = \frac{1}{\varepsilon} X_1(1 - X_1) - \frac{\delta}{\beta X_2 + 1} X_1 + \eta \tag{4.7}$$

$$\frac{dX_2}{d\tau} = \frac{1}{\varepsilon} X_2(1 - X_2) - \frac{\delta}{\beta X_1 + 1} X_2 + \eta \tag{4.8}$$

In Supplementary Figure 2g, we used the model above and the criterion tested is Supplementary Equation 3.20. Multiple sets of initial densities are tested. Although we observed a decreasing accuracy with increasing noise, the prediction accuracy is robustly maintained (above 90%) even when the standard

deviation of noise reaches 50% of signal strength. The high maintenance of prediction accuracy is due to the stability of the mutualistic model. Because the two stable steady states exist on opposite sides of the separatrix, only when noise pushes densities across the separatrix (against the vector field), the outcome of the system will be altered. Otherwise, noise will not influence the final steady state.

# 5. Calibration using SVM

We chose SVM because it is a well-established algorithm and it only requires information of the support vectors which are usually data points along the boundary to obtain a classification boundary. Note that other algorithms can also be implemented for the same purpose. For example, when predicting probability of coexistence, logistic regression can be more suitable to directly predict probability of coexistence. See the Supplementary Material for our MATLAB program which performs the calibration procedure using SVM.

## 1. Kernels

We used four types of kernels to cover different general shapes of the $B$ landscape:

Linear kernel: $$K_v\langle \boldsymbol{v_1}, \boldsymbol{v_2}\rangle = \boldsymbol{v_1}\cdot\boldsymbol{v_2} + k_\delta(\delta_1\cdot\delta_2) \quad (5.1)$$

Quadratic kernel: $$K_v\langle \boldsymbol{v_1}, \boldsymbol{v_2}\rangle = (\boldsymbol{v_1}\cdot\boldsymbol{v_2} + k_0)^2 + k_\delta(\delta_1\cdot\delta_2) \quad (5.2)$$

Cubic kernel: $$K_v\langle \boldsymbol{v_1}, \boldsymbol{v_2}\rangle = (\boldsymbol{v_1}\cdot\boldsymbol{v_2} + k_0)^3 + k_\delta(\delta_1\cdot\delta_2) \quad (5.3)$$

Sigmoidal kernel: $$K_v\langle \boldsymbol{v_1}, \boldsymbol{v_2}\rangle = \frac{(\boldsymbol{v_1}\cdot\boldsymbol{v_2})^2}{\|\boldsymbol{v_1}-\boldsymbol{v_2}\|^2+k_0} + k_\delta(\delta_1\cdot\delta_2) \quad (5.4)$$

Where in 5.4, $\|\boldsymbol{v_1}-\boldsymbol{v_2}\|^2 = \sum_i (v_{1i}-v_{2i})^2$.

These four kernels follow the same structure:

$$K\langle[\boldsymbol{v_1}, \delta_1], [\boldsymbol{v_2}, \delta_2]\rangle = K_v\langle \boldsymbol{v_1}, \boldsymbol{v_2}\rangle + k_\delta(\delta_1\cdot\delta_2),$$

Our procedure allows any kernel structure that is supplied by the user, so other customized kernels can also be used for the calibration. $k_\delta$ and $k_0$ are kernel parameters.

## 2. Standardizing input data

Standardizing input data is essential for robust estimation of $B(\boldsymbol{v})$. Before training the model with SVM, we first standardize the system variables $v$ and stress $\delta$ to mean 0 and variance 1.

$$\boldsymbol{v}^s = \frac{\boldsymbol{v} - mean(\boldsymbol{v})}{var(\boldsymbol{v})} \quad (5.5)$$

$$\boldsymbol{\delta}^s = \frac{\boldsymbol{\delta} - mean(\boldsymbol{\delta})}{var(\boldsymbol{\delta})} \quad (5.6)$$

For simplicity of presentation, $\boldsymbol{v}$ and $\boldsymbol{\delta}$ in section 5.3 represent the standardized inputs.

## 3. From SVM output to calibrated $\boldsymbol{B}(\boldsymbol{v})$

SVM will output a predictor that separates coexistence and collapse indicated in the $\boldsymbol{Y}$ vector. Our procedure requires that the two classes are represented by 1 and -1 in the $\boldsymbol{Y}$ vector. Using kernels 5.1-5.4, we can write the predictor in the general form:

$$f([\boldsymbol{v}, \delta]) = \sum_i \alpha_i y_i K_v\langle \boldsymbol{v_i}, \boldsymbol{v}\rangle + k_\delta \delta \sum_i \alpha_i y_i \delta_i + \lambda_0 \quad (5.7)$$

The predictor $f([\boldsymbol{v}, \delta])$ is a function of test variables $\boldsymbol{v}$ and $\delta$. Where values of $\boldsymbol{v}$ and $\delta$ for a new observation can be plugged in and a positive value of $f([\boldsymbol{v}, \delta])$ predicts coexistence and a negative value predicts collapse. In the predictor function, $k_\delta$ is a kernel parameter; $\alpha_i$ represents the coefficient associated with the $i^{th}$ observation that are solved by the SVM algorithm; $\lambda_0$ represents the bias that is optimized by the algorithm. $\boldsymbol{v}_i$ and $\delta_i$ are the normalized input data for observation $i$ and $y_i$ is the label indicating coexistence or collapse (1 or -1) for observation $i$.

If we impose $B = \delta$ at $f([\boldsymbol{v}, \delta]) = 0$, we can obtain a transformed function of $B$ in terms of input data:

$$B_0(\boldsymbol{v}) = \frac{-\sum_i \alpha_i y_i K_x\langle \boldsymbol{v}_i, \boldsymbol{v}\rangle - \lambda_0}{k_\delta \sum_i \alpha_i y_i \delta_i} \tag{5.8}$$

$B_0(\boldsymbol{v})$ can sometimes have the wrong directionality, meaning that $B/\delta > 1$ is associated with collapse and $B/\delta < 1$ is associated with coexistence. We identify these cases by calculating

$$\sum_1^n y_i \cdot sign(B_i - \delta_i) \quad , B_i = B_0(\boldsymbol{v_i}) \tag{5.9}$$

If the above expression is negative (when the trained boundary has a learning accuracy greater than 50%), the calibrated $B_0$ has a wrong directionality. Flipping the $B$ landscape is then required, and it is done by assuming:

$$-\left(\frac{B_0(\boldsymbol{v})}{\delta} - 1\right) = \frac{B^{flipped}(\boldsymbol{v})}{\delta} - 1 \tag{5.10}$$

Thus,

$$B^{flipped} = 2\delta - B_0(\boldsymbol{v}) \tag{5.11}$$

We name the output $B$ landscape with properly adjusted directionality $B'(v)$. This landscape $B'(v)$ describes the shape of the final calibrated $B(\boldsymbol{v})$ but still needs to be scaled back according to the mean and variance of the original $\boldsymbol{\delta}$ measurements. Thus,

$$B(\boldsymbol{v}) = B'(\boldsymbol{v}) \cdot var(\boldsymbol{\delta}) + mean(\boldsymbol{\delta}) \tag{5.12}$$

## 4. Cross validation and bootstrapping

All the cross validations in this work are 10-fold cross validations. The cross-validation accuracy is represented by the average values. Bootstrapping is used to evaluate the degree of variation of quantified $B$. The same number of data points as the input data is randomly sampled from the input data with replacement. We performed bootstrapping for 500 times. The variance and relative standard deviation (RSD) are then calculated based on the 500 bootstrapped $B(\boldsymbol{v})$ quantified with 500 sets of sampled training data. The mean cross validation accuracy, the bootstrapped variance and relative standard deviation are then used to evaluate the accuracy of the $B(\boldsymbol{v})$ outputs.

## 5. Twenty sets of simulations to establish and test the calibration procedure

To establish the calibration process, we first developed the method with simulated data where the true $B(\boldsymbol{v})$ are known. This allows us to evaluate calibration results against the ground truth. We used the model

presented in Supplementary Equations 3.22-23 to conduct the data for this analysis. Using $X_1$ as the reference population, the $B(\boldsymbol{v})$ can be expressed as:

$$B(\boldsymbol{\theta}) = \frac{\rho\left(\beta_1 + \frac{1}{\theta_1} + 1\right)^2}{4\beta_1\varepsilon_1\left(\frac{\rho}{\theta_1} + \theta_2\right)}, \theta_1 = \frac{\beta_2}{\beta_1}, \theta_2 = \frac{\varepsilon_2\delta_2}{\varepsilon_1\delta_1} \quad (5.13)$$

However, depending on how system variables change the model parameters ($\boldsymbol{\theta}(\boldsymbol{v})$) in the system variable space, the true $B(\boldsymbol{\theta}(\boldsymbol{v}))$ can exhibit diverse shapes. With $v_1, v_2 \in [0,1]$, we constructed 20 arbitrary sets of equations with different underlying functions. We also made sure the generated data have around 50:50 split of coexistence and collapse in the simulated results to reduce bias in the input data. This 50:50 split constraint also applies to all the other simulated data. Note that these functions are arbitrary and only serve the purpose of generating true $B(\boldsymbol{v})$ that has various underlying functions. The data generated using these 20 models are included in the Supplementary Software.

The 20 sets of $\boldsymbol{\theta}(\boldsymbol{v})$ are grouped into 5 types with each type containing 4 different examples. The 5 types of functions that describe $\boldsymbol{\theta}(\boldsymbol{v})$ include linear, quadratic, cubic, and Hill equation and another type of functions that are a mixture of the previous four types. As examples, the following are four sets of $\boldsymbol{\theta}(\boldsymbol{v})$ used to generate simulation results shown in Supplementary Figure 4b.

| | Linear | Quadratic |
|---|---|---|
| $\beta_1 =$ | $10v_1 - 2v_2 + 7$ | $-3.5(v_1 + 0.2)^2 - 5(v_2 - 0.2)^2 + 9$ |
| $\beta_2 =$ | $-5v_1 + 18v_2 + 6$ | $4(v_1 - 0.2)^2 + 5(v_2 - 0.2)^2 + 6.25$ |
| $\delta_1 =$ | $0.2v_1 + 0.4v_2 + 1.0$ | $0.07(v_1 - 0.2)^2 + 0.05(v_2 + 0.2)^2 + 1.09$ |
| $\delta_2 =$ | $-0.5v_1 + 2v_2 + 1.7$ | $0.05(v_1 + 0.1)^2 + 0.1(v_2 + 0.2)^2 + 1.3$ |
| $\varepsilon_1 =$ | $0.2v_1 + 0.1v_2 + 1$ | $0.05(v_1 + 0.5)^2 + 0.2(v_2)^2 + 1.1$ |
| $\varepsilon_2 =$ | $0.05v_1 + 0.1v_2 + 1$ | $0.18(v_1 + 0.2)^2 + 0.6(v_2 + 0.1)^2 + 0.9$ |

| | Cubic |
|---|---|
| $\beta_1 =$ | $0.2{v_1}^3 - 5(v_1 - 1)^2 + 5 + 0.2{v_2}^3 - 5(v_2 + 0.2)^2 + 10$ |
| $\beta_2 =$ | $0.16{v_1}^3 + 4(v_1 + 0.5)^2 - 0.1{v_2}^3 - 0.25(v_2)^2 + 3.5$ |
| $\delta_1 =$ | $0.28{v_1}^3 + 0.14(v_1)^2 - 0.12{v_2}^3 - 0.06(v_1 - 0.2)^2 + 1.2$ |
| $\delta_2 =$ | $0.04{v_1}^3 + 0.12(v_1 + 0.1)^2 + 0.01{v_2}^3 - 0.25(v_2 + 0.2)^2 + 4.95$ |
| $\varepsilon_1 =$ | $0.16{v_1}^3 + 0.02(v_1 - 0.3)^2 + 0.003{v_2}^3 + 0.06(v_2 + 0.2)^2 + 1.22$ |
| $\varepsilon_2 =$ | $-0.28{v_1}^3 + 0.14(v_1)^2 + 0.28 + 0.12{v_2}^3 - 0.06(v_2 - 0.2)^2 + 0.9$ |

| | Hill equation |
|---|---|
| $\beta_1 =$ | $10\frac{{v_1}^2}{{v_1}^2 + 0.5^2} - 6\frac{v_2}{v_2 + 1} + 12$ |
| $\beta_2 =$ | $2\frac{v_1}{v_1 + 1} - 5\frac{v_2}{v_2 + 1} + 9$ |
| $\delta_1 =$ | $0.4\frac{{v_1}^3}{{v_1}^3 + 0.5^3} + 0.2\frac{v_2}{v_2 + 1} + 1.01$ |

| | |
|---|---|
| $\delta_2 =$ | $0.1\frac{{v_1}^2}{{v_1}^2+1^2}+0.05\frac{{v_2}^2}{{v_2}^2+0.3^2}+1.2$ |
| $\varepsilon_1 =$ | $0.1\frac{{v_1}^5}{{v_1}^5+0.8^5}+0.2\frac{{v_2}^4}{{v_2}^4+1^4}+1$ |
| $\varepsilon_2 =$ | $0.2\frac{{v_1}^5}{{v_1}^5+0.5^5}+0.1\frac{{v_2}^2}{{v_2}^2+0.2^2}+1$ |

The training data are $10 \times 10$ on the $v_1, v_2$ space. $R^2$ between calibrated $B$ landscape ($B_c$) and the true landscape ($B_t$) is calculated by first performing a least square linear fit of $B_t$ with $B_c$. This fitting process aims to get a linear transformation of $B_c$ that conforms with the scale of $B_t$ while maintaining the shape of $B_c$. The absolute scale of the calibrated $B(\boldsymbol{v})$ is less crucial than its shape, and an absolute scale is also challenging to obtain.

$$B_c' = aB_c + b \tag{5.14}$$

Then $R^2$ is then calculated by:

$$1 - \frac{\sum(B_c' - B_t)^2}{\sum(B_t - \overline{B_t})^2} \tag{5.15}$$

## 6. Calibration with simulated data with *unknown* $B(\boldsymbol{\theta})$*:* an example

We want to test whether we can apply the calibration procedure to data that are generated by an arbitrary mutualism model. The specific model structure we used to generate data in Figure 2C is:

$$\frac{dX_1}{d\tau} = \frac{1}{\varepsilon_1\left(1+\frac{X_2}{X_2+0.2}\right)}X_1(1-X_1-X_2) - \frac{\delta_1}{{\beta_2}^3{X_2}^3+1}X_1 \tag{5.16}$$

$$\frac{dX_2}{d\tau} = \frac{1.5}{\varepsilon_2}X_2(1-0.8X_1-X_2) - \frac{\delta_2}{{\beta_1}^2{X_1}^2+1}X_2 \tag{5.17}$$

The analytical solution of this model cannot be expressed in a simple and explicit form. However, using simulated data, we can obtain an empirical function of $B$ on the system variable space that allow further prediction in the system variable space. The simulations are done using initial densities of [0.01, 0.01] for 10 unit-time.

To get the probability of coexistence, we ran the model 100 times with varying ratios of initial density while keeping the total initial density constant as 0.02. The density for a population is varied in a linear fashion from 0 to 0.02 and we terminated the simulation after 10 unit-time.

To calculate how well the systems can resist cheater exploitation, we modified the Supplementary Equations 5.16-5.17 to account for the emergence of cheaters:

$$\frac{dX_1}{d\tau} = \frac{1}{\varepsilon_1\left(1+\frac{X_2}{X_2+0.2}\right)}X_1(1-X_1-X_2-X_{1c}-X_{2c}) - \frac{\delta_1}{{\beta_2}^3{X_2}^3+1}X_1 - \varphi X_1 \tag{5.18}$$

$$\frac{dX_2}{d\tau} = \frac{1.5}{\varepsilon_2} X_2(1 - 0.8X_1 - X_2 - X_{1c} - X_{2c}) - \frac{\delta_2}{{\beta_1}^2 {X_1}^2 + 1} X_2 - \varphi X_2 \quad (5.19)$$

$$\frac{dX_{1c}}{d\tau} = X_1(1 - X_1 - X_2 - X_{1c} - X_{2c}) - \frac{\delta_1}{{\beta_2}^3 {X_2}^3 + 1} X_1 + \varphi X_1 \quad (5.20)$$

$$\frac{dX_{2c}}{d\tau} = 1.5\, X_2(1 - 0.8X_1 - X_2 - X_{1c} - X_{2c}) - \frac{\delta_2}{{\beta_1}^2 {X_1}^2 + 1} X_2 + \varphi X_2 \quad (5.21)$$

The simulations were done with $\varphi = 10^{-3}$, which is the transition rate from cooperators to cheaters. The initial densities are [0.01, 0.01, 0, 0] for $X_1$, $X_2$, $X_{1c}$, and $X_{2c}$, respectively. The simulations were terminated after 1300 unit-time.

## 7. Detailed graphical representation of the calibration procedure

Using the data generated using Supplementary Equations 5.16-5.17 as an example, the following figure shows a step-by-step graphical representation of the calibration procedure. The same procedure is applied to other simulated and experimental data. The different sets of data only differ in terms of how they are generated and what $v_1$ and $v_2$ correspond to.

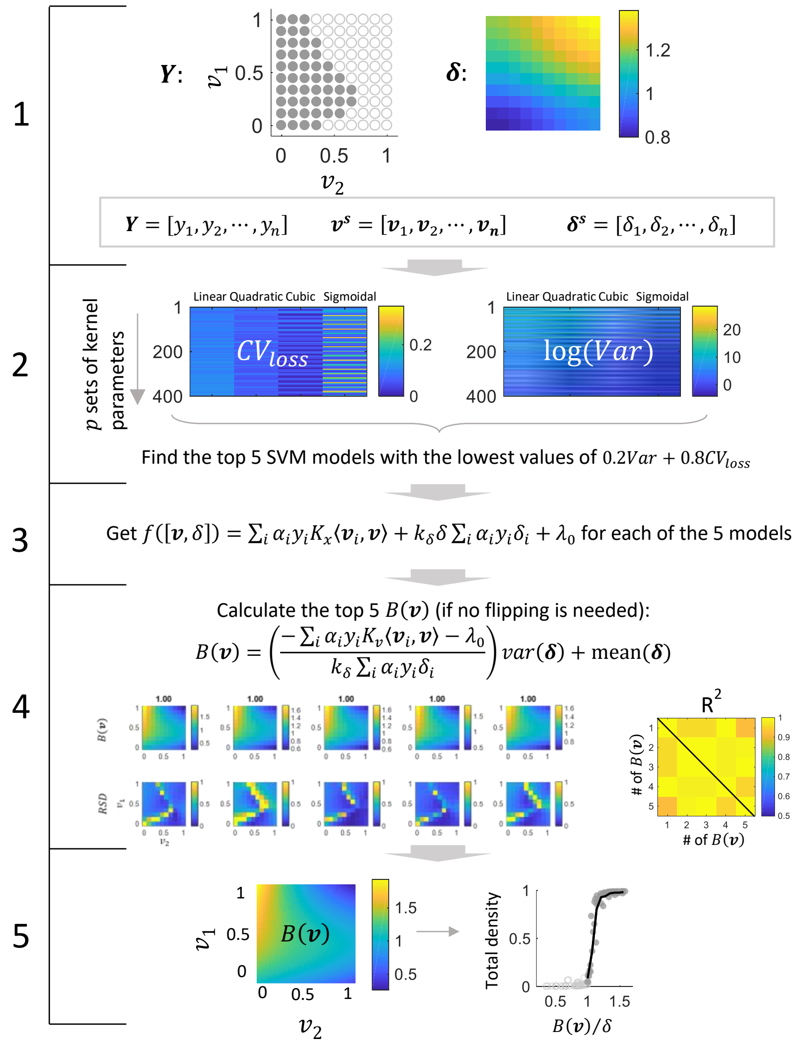

1. Data collection and preprocessing: Collect the measurements of coexistence vs. collapse ($\boldsymbol{Y}$) and stress ($\delta$) on the domain of $\boldsymbol{v}$. Above a certain threshold of total final density, assign $\boldsymbol{Y}$ a value of 1 and assign -1 if total final density is below this threshold. Standardize the values of $\delta$ and $v$ values to have mean of 0 and standard deviation of 1 and name the normalized vectors $\boldsymbol{\delta^s}$ and $\boldsymbol{v^s}$. For simplicity of presentation, the following $\boldsymbol{v}$ and $\boldsymbol{\delta}$ are standardized. In this example, we have 100 observations ($n =$ 100, 10 by 10) and two dimensions of $\boldsymbol{v}$ ($\boldsymbol{v_i} = (v_{i1}, v_{i2})$).
2. Pick the top SVM models: Using $\boldsymbol{Y}$, $\boldsymbol{\delta}$ and $\boldsymbol{v}$ as inputs, generate 1600 different SVM models, each containing one of the four kernel functions (linear, quadratic, cubic, and sigmoidal, see Supplementary Note 5.7) and one of 400 kernel parameter sets. For each kernel parameter, select values within a predefined range. The exact number of sets of kernel parameters used is not critical and can be adjusted depending on available computational resources.
   1) Calculate $CV_{loss}$: Each SVM model can then predict the coexistence or collapse for each combination of $\delta$ and $\boldsymbol{v}$ values. These predictions are used to calculate the average 10-fold cross-validation loss ($CV_{loss}$).
   2) Calculate $Var$: To calculate the variance for each SVM model, sample the 100 observations 100 times with replacement and calculate a single instance of $B(v)$. Repeat this process 500 times to obtain a distribution of 500 bootstrapped $B(v)$. Using this distribution, we can calculate the mean variance ($Var$) for each of the 1600 SVM models.
   3) Calculate $0.2Var + 0.8CV_{loss}$: We can empirically examine the top five SVM models, which were select by the lowest $0.2Var + 0.8CV_{loss}$ values. The weights of $Var$ and $CV_{loss}$ were determined empirically using simulated data (see Supplementary Figure 4c).
3. Train the top SVM models: Train each of the five SVM models with normalized input data to obtain the function that describes the boundary between the two classes. The functions can be expressed as:

$$f([\boldsymbol{v}, \delta]) = \sum_i \alpha_i y_i K_v \langle \boldsymbol{v}_i, \boldsymbol{v} \rangle + k_\delta \delta \sum_i \alpha_i y_i \delta_i + \lambda_0$$

   $\alpha_i$ is the weight of observation $i$, and $\lambda_0$ is the bias term for the SVM model. Both $\alpha_i$ and $\lambda_0$ are optimized by the SVM algorithm. $K_v$ is the kernel function; $k_\delta$ is a kernel parameter; $y_i$, $\boldsymbol{v}_i$, and $\delta_i$ are input values for observation $i$. $\boldsymbol{v}$ and $\delta$ are independent variables of the function. A positive value of $f([\boldsymbol{v}, \delta])$ for a new set of $\delta$ and $\boldsymbol{v}$ predicts coexistence and a negative value predicts collapse.
4. Quantify the top $B(v)$ and assess its reliability: Quantify $B(\boldsymbol{v})$ from each SVM model by imposing $B(\boldsymbol{v}) = \delta$ at $f([\boldsymbol{v}, \delta]) = 0$. If $B(\boldsymbol{v})$ has the correct directionality, get the function of $B(\boldsymbol{v})$ by calculating

$$B(\boldsymbol{v}) = \left( \frac{-\sum_i \alpha_i y_i K_v \langle \boldsymbol{v}_i, \boldsymbol{v} \rangle - \lambda_0}{k_\delta \sum_i \alpha_i y_i \delta_i} \right) \cdot var + mean$$

   $var$ and $mean$ are calculated using the original $\boldsymbol{\delta}$ measurements. See Supplementary Note 5.3. for how to adjust for the directionality of $B(\boldsymbol{v})$.
   1) Quantify relative standard deviations (RSD) of top $B(\boldsymbol{v})$: The sampling process is the same as step 2.2). Iterate 10 times to get 10 bootstrapped $B(\boldsymbol{v})$ to quantify an RSD value on each $(v_1, v_2)$ pair and construct a variability landscape of $B(\boldsymbol{v})$. The title of each $B(\boldsymbol{v})$ is the cross-validation accuracy of the model (1.00 corresponds to 100%).

2) Quantify pairwise consistencies of the top 5 models: To get $R^2$ of any two $B(\boldsymbol{v})$, the two scales of $B(\boldsymbol{v})$ are unified by first using linear fitting to adjust one $B(\boldsymbol{v})$ to conform to the scale of the other $B(\boldsymbol{v})$. $R^2$ is then calculated using the two $B(\boldsymbol{v})$ that have adjusted scales.
3) Pick the best $B(\boldsymbol{v})$: The first $B(\boldsymbol{v})$ is picked in this case since all top 5 have similar RSD and are all highly consistent.

5. Use $B(v)$ in downstream predictions: Use the best $B(v)$ to construct the metric $B(v)/\boldsymbol{\delta}$. We can then test whether $B(v)/\boldsymbol{\delta}$ is indeed positively related to the final total density in this example.

# 6. Framework generality verified by complex mutualistic systems

## 1. A 5-mutualist system

The data presented in Figure 4a and Supplementary Figure 9a are generated with the following mutualism model based on the general model structure presented in Supplementary Equation 3.39:

$$\frac{dX_1}{d\tau} = \frac{1}{\varepsilon_1} X_1 \left(1 - \sum_{k=1}^{5} X_k\right) - \frac{\delta_1}{\sum_{k\neq 1}^{n} \beta_k X_k + 1} X_1 \qquad (6.1)$$

$$\frac{dX_2}{d\tau} = \frac{1.1}{\varepsilon_2} X_2 \left(1 - \sum_{k=1}^{5} X_k\right) - \frac{\delta_2}{\sum_{k\neq 2}^{n} \beta_k X_k + 1} X_2 \qquad (6.2)$$

$$\frac{dX_3}{d\tau} = \frac{1.2}{\varepsilon_3} X_3 \left(1 - \sum_{k=1}^{5} X_k\right) - \frac{\delta_3}{\sum_{k\neq 3}^{n} \beta_k X_k + 1} X_3 \qquad (6.3)$$

$$\frac{dX_4}{d\tau} = \frac{1.3}{\varepsilon_4} X_4 \left(1 - \sum_{k=1}^{5} X_k\right) - \frac{\delta_4}{\sum_{k\neq 4}^{n} \beta_k X_k + 1} X_4 \qquad (6.4)$$

$$\frac{dX_5}{d\tau} = \frac{1.4}{\varepsilon_5} X_5 \left(1 - \sum_{k=1}^{5} X_k\right) - \frac{\delta_5}{\sum_{k\neq 5}^{n} \beta_k X_k + 1} X_5 \qquad (6.5)$$

All the parameters are linear combinations of $v_1, v_2 \in [0,1]$ and the coefficients are randomly generated. The initial densities are [0.001, 0.001, 0.001, 0.001, 0.001] for $X_1$ to $X_5$ respectively. The simulations were terminated after 5 unit-time.

## 2. The experimental 3-member mutualistic systems

The calibration shown in Figure 4b is done on all 384 systems with triplicates that alternatively use each strain in a system as the reference strain. The $\delta$ measurements used in this calibration are assumed to be the same as the measurements presented in Figure 3j and Supplementary 8b with the pairwise systems.

## 3. A mutualistic system in an oscillatory environment

The oscillatory signal is implemented as square pulses, where

$$S = \begin{cases} I, & \lceil \tau/L \rceil \text{ is odd} \\ 0, & \lceil \tau/L \rceil \text{ is even} \end{cases} \qquad (6.6)$$

$\tau$ is time; $L$ is the duration of the pulses and the duration between the end of one pulse and the start of the next pulse; $I$ is the intensity of the signal. Throughout the simulation, $\delta_1$, $\delta_2$, $\beta_1$, and $\beta_2$ are affected by this oscillating signal, where

$$\delta_1 = \delta_2 = 0.8 + S \tag{6.7}$$

$$\beta_1 = \beta_2 = 2(1 + S) \tag{6.8}$$

To simulate the data, we used a logarithm scale that vary $L$ from $10^{-2}$ to $10^{2}$ and vary $I$ from $10^{-1}$ to $10^{1}$. The model used to generate the set of data in Figure 4c and the left column of Supplementary Figure 10 is presented in Supplementary Equations 3.22-3.23, where $\rho = 1.5$. The initial densities used are [0.2, 0.2] for $X_1$ and $X_2$ respectively. The simulations are performed for 500 unit-time. The theoretical $B(\theta(\boldsymbol{v}))$ is calculated using the criterion 3.32 with $\delta_1 = \delta_2 = 0.8 + I$ and $\beta_1 = \beta_2 = 2(1 + I)$.

## 4. A mutualistic system cohabiting with other populations

To generate simulated data presented in Figure 4d and the right column of Supplementary Figure 10, we used the following arbitrary 7-population model, where $X_1$ and $X_2$ are mutualistic and their population dynamics are modulated by the other 5 populations.

$$\frac{dX_1}{d\tau} = \frac{1}{\varepsilon} X_1 \left(1 - \sum X_i\right) - \frac{\delta}{\beta X_2 + \beta_{51} X_5 + 1} X_1 - X_6 X_1 \tag{6.9}$$

$$\frac{dX_2}{d\tau} = \rho \frac{1}{\varepsilon} X_2 \left(1 - \sum X_i\right) - \frac{\delta}{\beta X_1 + \beta_{62} X_6 + 1} X_2 - X_5 X_2 \tag{6.10}$$

$$\frac{dX_3}{d\tau} = X_3 \left(1 - \sum X_i\right) - \frac{\delta_3}{\beta_{13} X_1 + \beta_{23} X_2 + 1} X_3 \tag{6.11}$$

$$\frac{dX_4}{d\tau} = X_4 \left(1 - \sum X_i\right) - \frac{\delta_4}{\beta_{34} X_3 + \beta_{23} X_2 + 1} X_4 \tag{6.12}$$

$$\frac{dX_5}{d\tau} = X_5 \left(1 - 2 \sum X_i\right) \tag{6.13}$$

$$\frac{dX_6}{d\tau} = X_6 \left(1 - 2 \sum X_i\right) \tag{6.14}$$

$$\frac{dX_7}{d\tau} = X_7 \left(1 - 3.3 \sum X_i\right) + \frac{X_1}{X_1 + 0.5} X_7 - X_2 X_7 \tag{6.15}$$

In the simulation, only $\delta$ and $\beta$ are modulated by system variables $v_1, v_2 \in [0,1]$, while other parameters were kept constants.

$$\delta = 0.5 v_1 + 1.2 v_2 + 0.8 \tag{6.16}$$

$$\beta = -15 v_1 - 10 v_2 + 30 \tag{6.17}$$

The initial densities are [1, 1, 1, 1, 1, 1, 1] for $X_1$ to $X_7$ respectively. The simulations were performed for 5000 unit-time. The theoretical $B(\theta(\boldsymbol{v}))/\delta$ in the right panel of Supplementary Figure 10a is directly calculated using criterion expressed in Supplementary Equation 3.32.

# Supplementary References


1 Bascompte, J., Jordano, P. & Olesen, J. M. Asymmetric coevolutionary networks facilitate biodiversity maintenance. *Science* **312**, 431-433, doi:10.1126/science.1123412 (2006).

2 Allesina, S. & Tang, S. Stability criteria for complex ecosystems. *Nature* **483**, 205-208 (2012).

3 Coyte, K. Z., Schluter, J. & Foster, K. R. The ecology of the microbiome: Networks, competition, and stability. *Science* **350**, 663-666, doi:10.1126/science.aad2602 (2015).

4 May, R. M. Citation Classic - Stability and Complexity in Model-Ecosystems. *Cc/Agr Biol Environ*, 22-22 (1988).

5 DeAngelis, D. L., Post, W. M. & Travis, C. C. *Positive feedback in natural systems*. (Springer-Verlag, 1986).

6 Mougi, A. & Kondoh, M. Diversity of Interaction Types and Ecological Community Stability. *Science* **337**, 349-351, doi:10.1126/science.1220529 (2012).

7 Thebault, E. & Fontaine, C. Stability of Ecological Communities and the Architecture of Mutualistic and Trophic Networks. *Science* **329**, 853-856, doi:10.1126/science.1188321 (2010).

8 Stachowicz, J. J. Mutualism, facilitation, and the structure of ecological communities. *Bioscience* **51**, 235-246, doi:Doi 10.1641/0006-3568(2001)051[0235:Mfatso]2.0.Co;2 (2001).

9 Bastolla, U. *et al.* The architecture of mutualistic networks minimizes competition and increases biodiversity. *Nature* **458**, 1018-1020, doi:10.1038/nature07950 (2009).

10 Addicott, J. F. Stability properties of 2-species models of mutualism: Simulation studies. *Oecologia* **49**, 42-49, doi:10.1007/BF00376896 (1981).

11 Momeni, B., Xie, L. & Shou, W. Lotka-Volterra pairwise modeling fails to capture diverse pairwise microbial interactions. *eLIFE* (2017).

12 Holland, J. N. & DeAngelis, D. L. Interspecific population regulation and the stability of mutualism: fruit abortion and density-dependent mortality of pollinating seed-eating insects. *Oikos* **113**, 563-571, doi:DOI 10.1111/j.2006.0030-1299.14430.x (2006).

13 Holland, J. N., DeAngelis, D. L. & Bronstein, J. L. Population dynamics and mutualism: Functional responses of benefits and costs. *Am Nat* **159**, 231-244 (2002).

14 Garcia-Algarra, J., Galeano, J., Pastor, J. M., Iriondo, J. M. & Ramasco, J. J. Rethinking the logistic approach for population dynamics of mutualistic interactions. *Journal of theoretical biology* **363**, 332-343, doi:10.1016/j.jtbi.2014.08.039 (2014).

15 Holland, J. N. & DeAngelis, D. L. A consumer-resource approach to the density-dependent population dynamics of mutualism. *Ecology* **91**, 1286-1295 (2010).

16 Stanton, M. L. Interacting guilds: Moving beyond the pairwise perspective on mutualisms. *Am Nat* **162**, S10-S23 (2003).

17 Munns, R. Comparative physiology of salt and water stress. *Plant Cell and Environment* **25**, 239-250, doi:DOI 10.1046/j.0016-8025.2001.00808.x (2002).

18 Schimel, J., Balser, T. C. & Wallenstein, M. Microbial stress-response physiology and its implications for ecosystem function. *Ecology* **88**, 1386-1394, doi:Doi 10.1890/06-0219 (2007).

19 Hoegh-Guldberg, O. *et al.* Coral reefs under rapid climate change and ocean acidification. *Science* **318**, 1737-1742, doi:10.1126/science.1152509 (2007).

20 Finkel, T. & Holbrook, N. J. Oxidants, oxidative stress and the biology of ageing. *Nature* **408**, 239-247, doi:Doi 10.1038/35041687 (2000).

21 Hoek, T. A. *et al.* Resource Availability Modulates the Cooperative and Competitive Nature of a Microbial Cross-Feeding Mutualism. *PLoS biology* **14**, doi:ARTN e1002540

10.1371/journal.pbio.1002540 (2016).

22 Brown, B. E. Coral bleaching: causes and consequences. *Coral Reefs* **16**, S129-S138 (1997).

23 Webster, N. S., Cobb, R. E. & Negri, A. P. Temperature thresholds for bacterial symbiosis with a sponge. *Isme Journal* **2**, 830-842, doi:10.1038/ismej.2008.42 (2008).

24 Holcomb, M., McCorkle, D. C. & Cohen, A. L. Long-term effects of nutrient and CO2 enrichment on the temperate coral Astrangia poculata (Ellis and Solander, 1786). *J Exp Mar Biol Ecol* **386**, 27-33, doi:10.1016/j.jembe.2010.02.007 (2010).
25 Cipollini, D., Rigsby, C. M. & Barto, E. K. Microbes as Targets and Mediators of Allelopathy in Plants. *J Chem Ecol* **38**, 714-727, doi:10.1007/s10886-012-0133-7 (2012).
26 Bronstein, J. L. *Mutualism*. First edition. edn, (Oxford University Press, 2015).
27 Balagadde, F. K. *et al.* A synthetic Escherichia coli predator-prey ecosystem. *Molecular Systems Biology* **4**, doi:ARTN 187

10.1038/msb.2008.24 (2008).
28 Neuhauser, C. & Fargione, J. E. A mutualisim-parasitism continuum model and its application to plant-mycorrhizae interactions. *Ecol Model* **177**, 337-352, doi:10.1016/j.ecolmodel.2004.02.010 (2004).
29 Yamamura, N. Evolution of mutualistic symbiosis: A differential equation model. *Res Popul Ecol* **38**, 211-218, doi:Doi 10.1007/Bf02515729 (1996).
30 Morris, W. F., Bronstein, J. L. & Wilson, W. G. Three-way coexistence in obligate mutualist-exploiter interactions: The potential role of competition. *Am Nat* **161**, 860-875, doi:Doi 10.1086/375175 (2003).
31 Kerner, A., Park, J., Williams, A. & Lin, X. N. A programmable Escherichia coli consortium via tunable symbiosis. *PloS one* **7**, e34032, doi:10.1371/journal.pone.0034032 (2012).
32 Rohr, R. P., Saavedra, S. & Bascompte, J. On the structural stability of mutualistic systems. *Science* **345**, 416-+, doi:10.1126/science.1253497 (2014).
33 Morris, W. F., Vazquez, D. P. & Chacoff, N. P. Benefit and cost curves for typical pollination mutualisms. *Ecology* **91**, 1276-1285 (2010).
34 Bertness, M. D. & Leonard, G. H. The role of positive interactions in communities: Lessons from intertidal habitats. *Ecology* **78**, 1976-1989 (1997).
35 Palmer, T. M. *et al.* Breakdown of an ant-plant mutualism follows the loss of large herbivores from an African Savanna. *Science* **319**, 192-195 (2008).
