# Supplementary material for: A unifying framework for interpreting and predicting mutualistic systems
Source: Nat Commun. 2019 Jan 16;10:242. doi: 10.1038/s41467-018-08188-5 (PMC6335432; doi:10.1038/s41467-018-08188-5)
Supplement: Supplementary file 2 — Description of Additional Supplementary Files [file 41467_2018_8188_MOESM2_ESM.docx]

**Description of Additional Supplementary Files**

File Name: Supplementary Movie 1

Description: This video demonstrates the relative position of the boundary surface (𝛿, 𝒗) = 0 and 5 observations in the schematic shown in Figure 2b. Input data in Figure 2b can be represented in a 3D space. The color of the dots and the z axis positions both indicate 𝛿 values. Coexistence is indicated by closed circles and collapse is indicated by open circles. The gray boundary surface separates coexistence and collapse. The surface is above observations that represent coexistence (𝐵 > 𝛿) and below observations that represent collapse (𝐵 < 𝛿). This surface can be directly interpreted as (𝒗) since 𝐹(𝛿, 𝒗) = 0 ⟹ 𝐹(𝐵, 𝒗) = 0 ⟹ 𝐵(𝒗) .

File Name: Supplementary Movies 2-4

Description: These 3 videos show the calibrated surface of (𝒗) relative to input data in 3D space. The blue surfaces are the boundary (𝛿, 𝒗) = 0 that separate coexistence and collapse. The surface is also equivalent to (𝒗). The indices on x and y axes in video 4 correspond to the same strain orders in Figure 3j.

File Name: Supplementary Software 1

Description: The Supplementary Software is in coded in MATLAB. The software is comprised of two parts. The first part contains the derivation process of coexistence criteria for all models presented in Supplementary Table 1 and 2. The second part contains the calibration procedure.
